# Wnt evolution and function shuffling in liberal and conservative chordate genomes

**DOI:** 10.1186/s13059-018-1468-3

**Published:** 2018-07-25

**Authors:** Ildikó M. L. Somorjai, Josep Martí-Solans, Miriam Diaz-Gracia, Hiroki Nishida, Kaoru S. Imai, Hector Escrivà, Cristian Cañestro, Ricard Albalat

**Affiliations:** 1 Biomedical Sciences Research Complex, School of Biology, University of St Andrews, North Haugh, St Andrews, KY16 9ST Scotland, UK; 2 Scottish Oceans Institute, School of Biology, University of St Andrews, East Sands, St Andrews, KY16 8LB Scotland, UK; 3 Departament de Genètica, , Microbiologia i Estadística, and Institut de Recerca de la Biodiversitat (IRBio), Universitat de Barcelona, Barcelona, Spain; 4 Department of Biological Sciences, Graduate School of Science, Osaka University, Toyonaka, Osaka 560-0043 Japan; 5 Sorbonne Universités, UPMC Univ Paris 06, CNRS, Biologie Intégrative des Organismes Marins (BIOM), Observatoire Océanologique, F-66650 Banyuls/Mer, France

**Keywords:** Wnt evolution, Genome stasis, Gene loss, Function shuffling, Chordate WntA, Ascidians, Vertebrates, Amphioxus

## Abstract

**Background:**

What impact gene loss has on the evolution of developmental processes, and how function shuffling has affected retained genes driving essential biological processes, remain open questions in the fields of genome evolution and EvoDevo. To investigate these problems, we have analyzed the evolution of the Wnt ligand repertoire in the chordate phylum as a case study.

**Results:**

We conduct an exhaustive survey of *Wnt* genes in genomic databases, identifying 156 *Wnt* genes in 13 non-vertebrate chordates. This represents the most complete *Wnt* gene catalog of the chordate subphyla and has allowed us to resolve previous ambiguities about the orthology of many *Wnt* genes, including the identification of *WntA* for the first time in chordates. Moreover, we create the first complete expression atlas for the Wnt family during amphioxus development, providing a useful resource to investigate the evolution of *Wnt* expression throughout the radiation of chordates.

**Conclusions:**

Our data underscore extraordinary genomic stasis in cephalochordates, which contrasts with the liberal and dynamic evolutionary patterns of gene loss and duplication in urochordate genomes. Our analysis has allowed us to infer ancestral Wnt functions shared among all chordates, several cases of function shuffling among *Wnt* paralogs, as well as unique expression domains for *Wnt* genes that likely reflect functional innovations in each chordate lineage. Finally, we propose a potential relationship between the evolution of *WntA* and the evolution of the mouth in chordates.

**Electronic supplementary material:**

The online version of this article (10.1186/s13059-018-1468-3) contains supplementary material, which is available to authorized users.

## Background

The era of comparative genomics is providing a new perspective on the evolution of living diversity by revealing an unexpected and significant amount of genetic complexity that already existed in ancestral organisms. This new perspective implies that evolutionary simplification—and not only complexification resulting from the acquisition of gene novelties [1] or from the co-option of pre-existing or duplicated genes for novel functions [2–5]—has been a prominent trend across the Tree of Life (reviewed in [6]). At the genetic level, simplification has often been accompanied by pervasive gene loss, providing an important source of genetic variation, in many cases even eliciting major evolutionary adaptive responses—“the less is more” principle [7]—(reviewed in [8]). However, understanding the significance of gene loss [8] and function shuffling among duplicated genes [9] on the generation of biodiversity, and especially their impact on the evolution of the genetic mechanisms of development of complex multicellular animals, is still a fundamental problem in evolutionary and developmental biology. To explore this problem, we focus here on the evolution of the Wingless/Wnt family in chordates as a paradigm; the Wnt family is among the best characterized of all metazoan gene families (reviewed in [8, 10, 11]) and plays conserved roles in fundamental developmental processes in all animals, including determination of the primary body axis, spatial cell patterning, cell fate specification, and cell proliferation and migration (reviewed in [11–13]).

The Wnt family encodes a set of secreted glycoprotein ligands that trigger a variety of signal transduction pathways to regulate gene transcription in target cells (e.g., [14]). The transduction of Wnt signaling can occur via two main pathways, the “canonical” and the “non-canonical”, although they are not mutually exclusive [11]. The “canonical” Wnt/β-catenin pathway (a.k.a. cell-fate pathway) is mediated by the stabilization and transport of β-catenin into the nucleus, where it binds to transcription factors that regulate the expression of Wnt target genes, and thus specify cellular fates. The various Wnt signaling pathways that act independently of β-catenin have been described as non-canonical and, despite their diverse functions, they can be broadly grouped into the so-called “Wnt cell polarity” pathway [11]. The ascription of each Wnt family member to a particular pathway is not straightforward and it largely depends on the ability of each ligand to modulate β-catenin availability or, alternatively, to mediate cell behaviors. From cnidarians to vertebrates, for instance, Wnt1 and Wnt3 have been generally considered to signal through the canonical pathway, while Wnt5 and Wnt11 have been typically assigned to the Wnt cell polarity pathway [15–17]. The fact, however, that certain Wnt ligands can be promiscuous and activate more than one pathway (e.g., [18]) makes it difficult to assign them to any specific group.

*Wnt* genes are a metazoan novelty [1] found from sponges to humans that duplicated and diversified into 13 subfamilies—Wnt1 to Wnt11, Wnt16, and WntA—before the bilaterian and cnidarian split [19, 20]. Large-scale phylogenetic and genomic analyses have revealed that several *Wnt* genes have been lost and retained during animal evolution [8, 10, 11, 21–23]. For instance, the gastropod *Patella vulgata* is the protostome that has suffered the greatest number of losses (9 out of 13, only preserving Wnt1, Wnt2, Wnt10, and WntA subfamilies), which is in stark contrast with the only two losses (Wnt3 and Wnt8) seen in another gastropod, *Lottia gigantea* [24, 25]. Other species such as *Drosophila melanogaster* and *Caenorhabditis elegans*, which have lost six (Wnt2 to 4, 11, 16, and A) and eight (Wnt1 to 3, 6 to 8, 11, and 16) subfamilies, respectively, make it evident that each animal lineage has shaped its own repertoire of *Wnt* genes. They also reveal that while many gene losses are recurrent and occurred independently in many lineages, others are ancestral and possibly important for the evolution of specific clades. For instance, the patchy pattern of absence/presence of *Wnt9* suggests that it has been lost at least eight times during the evolution of arthropods, annelids, platyhelminthes, and cnidarians, while the absence of *Wnt3* in all protostomes suggests an early loss of *Wnt3* in the stem protostome ancestor, likely affecting the evolution of the entire group (reviewed in [8, 10, 11]).

In contrast to protostomes, vertebrates appear refractory to the loss of entire Wnt subfamilies. They have preserved at least one member in 12 out of the 13 subfamilies, with WntA the only subfamily that has not been found so far in vertebrates [24]. However, whether the tendency to retain Wnt subfamilies is specific to vertebrates or rather is a feature shared by all chordates (i.e., vertebrates + urochordates + cephalochordates) remains unkown. In urochordates (tunicates), the Wnt repertoire remains unresolved: phylogenetic classifications of *Wnt* genes from partial studies in three ascidians species (i.e., *Halocynthia roretzi*, *Ciona robusta*, and *Botryllus schlosseri*) have resulted in many unascribed Wnt genes (referred to as “orphan” *Wnt* genes) and, in some cases, conflicting orthologies due to the high sequence divergence typical of these species [26–33]. In cephalochordates, so far only eight *Wnt* genes (*Wnt1*, *3*, *4*, *5*, *6*, *7*, *8*, *11*) have been studied in one amphioxus species, *Branchiostoma floridae* [34–39] (reviewed in [40]), of which five (*Wnt3*, *5*, *6*, *7*, and *8*) have been partially characterized in another, *B. lanceolatum* [41, 42]. Consequently, the taxonomic diversity of the analyzed urochordate and cephalochordate species has been too narrow, and the phylogenetic analysis of non-vertebrate chordate *Wnt* genes too ambiguous, to draw general conclusions about the evolution and function of orthologous Wnt subfamilies in the chordate phylum.

In order to provide a comprehensive view of the evolution of Wnt subfamilies in chordates, we have conducted an exhaustive survey of *Wnt* genes in genomic databases of ten ascidian (urochordate subphylum) and three amphioxus (cephalochordate subphylum) species and have generated the first complete atlas of developmental expression of the Wnt family in amphioxus. Our phylogenetic analysis represents, to our knowledge, the first fully resolved reconstruction of all Wnt subfamilies in the three chordate subphyla, resolving previous ambiguous or conflictive ascriptions of orthology. Our study reveals opposite trends in *Wnt* gene losses and retentions in cephalochordates and urochordates: while amphioxus shows a conservative pattern of evolution, retaining the complete ancestral repertoire of chordate Wnt subfamilies, ascidians in contrast reveal a dynamic pattern of evolution, with numerous gene losses and duplications. Our study also demonstrates for the first time the presence of *WntA* genes in chordates (both in cephalochordates and in urochordates), which implies that the absence of the WntA subfamily in vertebrates is not due to an ancestral loss in chordates as previously suggested, but to a specific gene loss occurring during the early evolution of vertebrates. Finally, our detailed atlas of *Wnt* expression in amphioxus, including the newly identified *WntA* genes in non-vertebrate chordates as well as several cases of “function shuffling” [9], allows us to evaluate the contributions of different Wnt subfamilies to the diversification of each chordate subphylum.

## Results

### The Wnt gene repertoire in non-vertebrate chordates

**Fig. 1:**
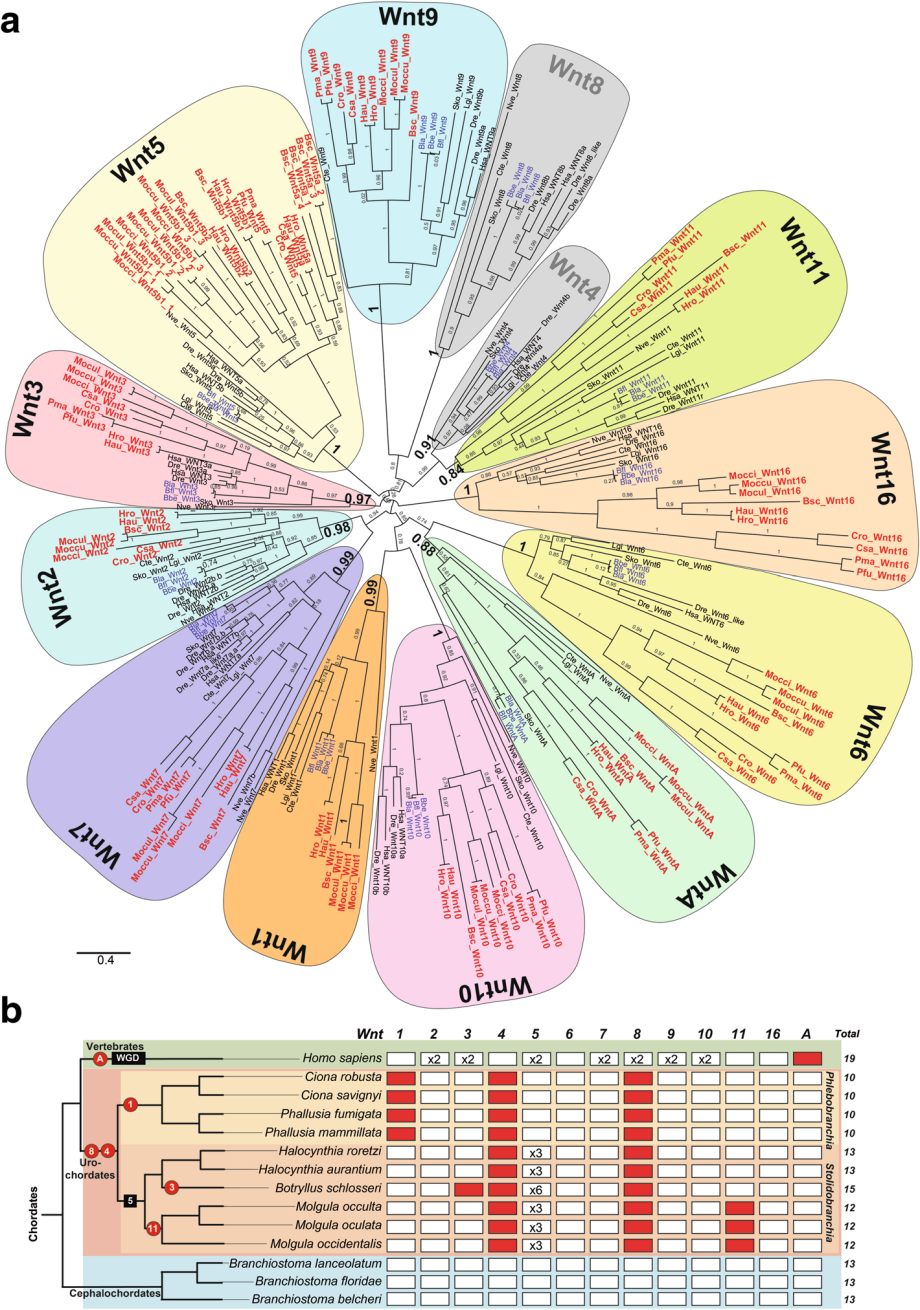
Wnt family evolution in chordates. **a** The ML phylogenetic tree reveals a conservative pattern of genomic evolution in cephalochordates (species names in *blue*), which preserve all 13 Wnt subfamilies, contrasting with the dynamic pattern of genomic evolution in urochordates (in *red*), which are characterized by several gene losses and duplications. The *scale bar* indicates amino acid substitutions. Values for the approximate likelihood ratio test (aLRT) are shown at nodes. Species are as follows. Chordate species: Urochordates: *Botryllus schlosseri* (*Bsc*), *Ciona savignyi* (*Csa*), *Ciona robusta* (*Cro*; formerly *Ciona intestinallis*), *Halocynthia roretzi* (*Hro*), *Halocynthia aurantium* (*Hau*), *Mogula occulta* (*Moccu*), *Mogula oculata* (*Mocul*), *Mogula occidentalis* (*Mocci*), *Phallusia fumigata* (*Pfu*), and *Phallusia mammillata* (*Pma*). Cephalochordates: *Branchiostoma belcheri* (*Bbe*), *Branchiostoma floridae* (*Bfl*), *Branchiostoma lanceolatum* (*Bla*). Vertebrates: *Danio rerio* (*Dre*), *Homo sapiens* (*Hsa*). Non-chordates species: hemichordate *Saccoglossus kowalevskii* (*Sko*), annelid *Capitella teleta* (*Cte*), mollusk *Lottia gigantea* (*Lgi*), and cnidarian *Nematostella vectensis* (*Nve*). **b** The *Wnt* gene catalog (*Wnt1–11, Wnt16* and *WntA*) present (*white squares*) or absent (*red squares*) in the three chordate subphyla, allowing inference of plausible events of gene losses (*red circles*; the number or letter inside the circle indicates the lost Wnt subfamily) and duplications (*black squares*; the number inside the square indicates the duplicated subfamily) during the evolution of different lineages. While some losses appear to be ancestral (e.g., *Wnt8* and *Wnt4* losses in stem urochordates), others only affected specific groups or species (e.g., *Wnt1* loss in *Phlebobranchia*, *Wnt11* in *Molgulas*, and *Wnt3* in *Botryllus*). In vertebrates, Wnt subfamilies expanded following the rounds of whole-genome duplications (*WGD*) that occurred during their early evolution [4], while in the rest of chordates all *Wnt* are conserved as a single copy, with the exception of *Wnt5* in Stolidobranchian ascidians, which has suffered multiple events of tandem gene duplications (see Additional file 1: Figure S1)

Our comprehensive survey of *Wnt* genes in genomic databases of 13 non-vertebrate chordate species—three cephalochordate species and ten urochordate species representing five ascidian families from two different orders (two solitary Cionidae and two solitary Ascidiidae within Phlebobranchia; and two solitary Pyuridae, three solitary Molgulidae, and one colonial Styelidae within Stolidobranchia)—identified 156 *Wnt* genes (Additional file 1: Table S1), which constitutes the first comprehensive catalog of *Wnt* genes in non-vertebrate chordates. Our phylogenetic analyses, which included a total of 247 Wnt sequences from 19 species (sequence alignment in Additional file 2) representing all major metazoan groups, from cnidarians to vertebrates, sorted the non-vertebrate chordate Wnt sequences into 13 monophyletic groups corresponding to the 13 known Wnt subfamilies (Fig. 1).

#### Conservative Wnt evolution in cephalochordates

Our phylogenetic analyses revealed that the three *Branchiostoma* species possess one ortholog for each of the 13 ancient Wnt subfamilies (Fig. 1a, b; see Additional file 2 for sequence alignment). Our results identified five *Wnt* genes that had not been analyzed in cephalochordates before and corroborated the orthology of eight previously described amphioxus *Wnt* genes [34–39, 41] (reviewed in [40]). We further extended our Wnt survey to the transcriptome project of *Asymmetron lucayanum*, a cephalochordate distantly related to the other *Branchiostoma* species [43]. We identified 13 Wnt sequences, mostly full length (Additional file 1: Table S1), each one orthologous to one of the 13 Wnt subfamilies (Additional file 1: Figure S4; see Additional file 3 for sequence alignment). The fact that all amphioxus Wnt orthologs form a single cluster nested within vertebrate and ambulacrarian sequences from each Wnt subfamily suggests that neither gene duplications nor gene losses affected the evolution of Wnt subfamilies in the cephalochordate subphylum. Our findings, therefore, reinforce the idea of genomic and morphological evolutionary stasis attributed to cephalochordate species [41, 44–48] (reviewed in [49]), in spite of divergence times estimated at over tens of millions of years ago [43, 50–53].

#### Liberal Wnt evolution with losses and duplications in ascidian urochordates

Our phylogenetic analyses provided the first fully resolved orthology of ascidian *Wnt* genes, allowing us to rename previously described genes still classified as “orphan *Wnts*” with unclear orthology, as well as to settle conflicting orthology assignments, probably caused by limited species sampling [29–32] (Fig. 1a and Additional file 1: Table S1). The phylogenetic tree showed long branches for ascidian *Wnt* genes, which rarely clustered as the sister group of vertebrate *Wnt* genes within each Wnt subfamily, as would be expected from their taxonomic relationships, likely due to artifactual “long-branch attraction” [54]. Our results showed that ascidians, in contrast to amphioxus, do not conserve the entire Wnt repertoire and have suffered various events of gene loss, as well as gene duplications (Fig. 1). While some of the losses appeared to be ancestral, resulting in the absence of Wnt subfamilies in all ascidian species, other losses and duplications affected different families and orders heterogeneously, suggesting a more dynamic evolution of *Wnt* genes in ascidians than in the conservative amphioxus. *Wnt4* and *Wnt8*, for instance, are absent in all analyzed species, plausibly due to two ancestral gene losses that occurred prior to the ascidian radiation, and therefore relevant to our understanding of the divergence in developmental mechanisms between ascidians and other chordates (Fig. 1b). On the other hand, *Wnt1* and *Wnt11* appear to be absent in species of the Phelobobranchia suborder and *Molgula* genus, respectively, while *Wnt3* is only absent in *B. schlosseri*. It seems, therefore, that loss of *Wnt* genes might have contributed to the genetic divergence between different groups or even single species of ascidians.

Our results, moreover, revealed that the Wnt5 subfamily in the Stolidobranchia order had experienced extensive amplification by gene duplication, affecting all six analyzed species (Fig. 1b). The fact that many of these *Wnt5* duplicates appeared to be located in the same genomic regions suggested that they originated by tandem gene duplications (Additional file 1: Figure S1). The complex phylogenetic reconstructions of the ascidian Wnt5 clade were difficult to interpret, suggesting either the occurrence of independent gene duplications in different lineages after events of speciation or, alternatively, ancestral *Wnt5* duplications in stem Stolidobranchia, followed by multiple gene losses and events of gene conversion (Additional file 1: Figure S1). In either case, the expansion of *Wnt5* in Stolidobranchia provides a singular case of Wnt subfamily amplification in non-vertebrate chordates, suggesting that the evolution of this order of ascidian species has been accompanied by a relaxation of the evolutionary constraints that maintain *Wnt5* genes as a single copy gene in other species. This may be linked to the recruitment of new *Wnt5* paralogs in biological innovations unique to this group of ascidians.

Overall, the dynamic pattern of gene losses and duplications of *Wnt* genes observed among different orders, families, or individual species of ascidians correlates with the burst of morphological diversification within the urochordate subphylum, contrasting with the conservative pattern of Wnt evolution and morphological stasis observed in cephalochordates.

#### WntA, lost and found in chordates

Our analysis also led to the identification of *WntA* genes in cephalochordates and urochordates (Fig. 1 and Additional file 1: Table S1). This finding was of special interest since *WntA* had previously been identified in cnidarians, protostomes, and ambulacrarian deuterostomes (e.g., in the hemichordate *Saccoglossus kowalevskii* and the echinoderm *Strongylocentrotus purpuratus*), but not in any chordate, leading to the suggestion that the WntA subfamily had been lost in stem chordates [10, 24]. The identification in this study of *WntA* genes in both cephalochordate and urochordate species implies, therefore, that *WntA* was present in the last common chordate ancestor, preserved in cephalochodates and urochordates, but specifically lost during the early evolution of the vertebrate lineage.

### The complete expression atlas of cephalochordate Wnt genes

**Fig. 2:**
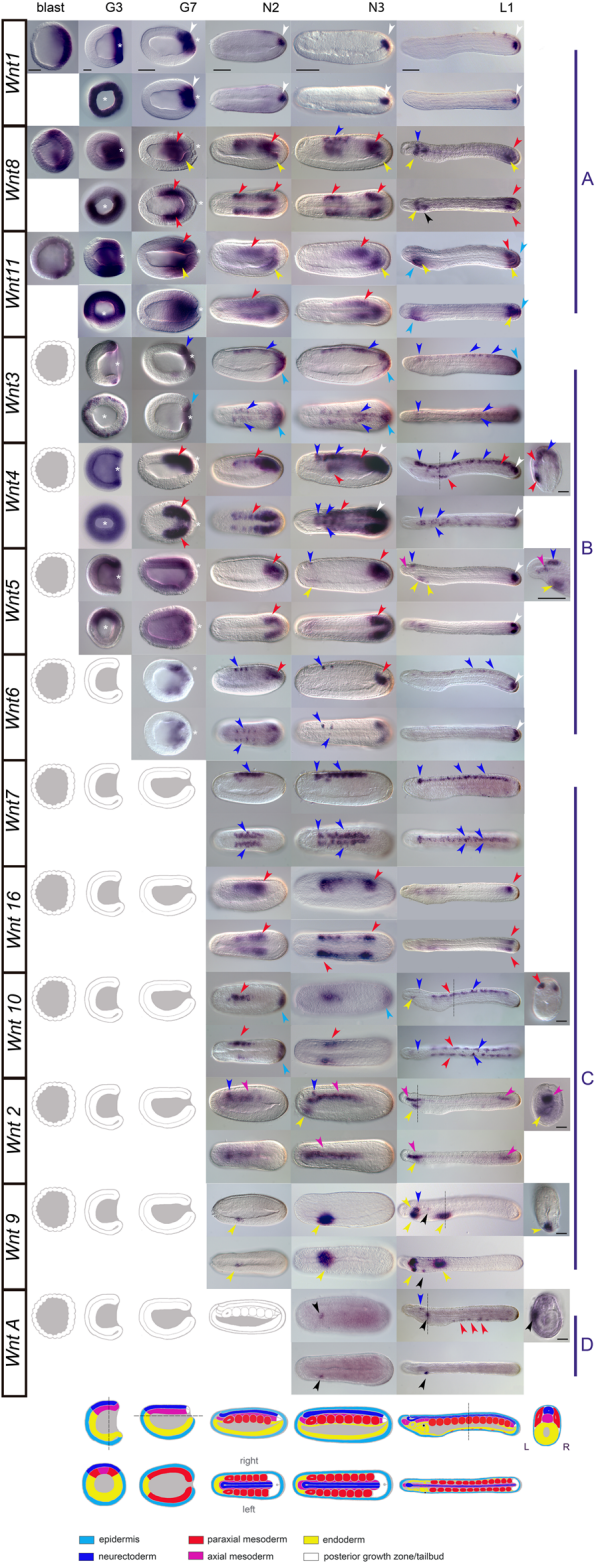
Complete atlas of *Wnt* expression in the cephalochordate *Branchiostoma lanceolatum*. Stage-matched expression patterns of 13 amphioxus *Wnt* genes reveal complex spatio-temporal choreography throughout embryogenesis. *Wnt* genes are ordered according to their developmental timing of expression: *Wnt1*, *Wnt8*, and *Wnt11* are first expressed in the blastula (class A genes); *Wnt3*, *Wnt4*, *Wnt5*, and *Wnt6* begin in the gastrula (class B genes); *Wnt7*, *Wnt16*, *Wnt10*, *Wnt2*, and *Wnt9* in the early neurula (N2; class C genes); and *WntA* in the mid-late neurula (N3 class D gene). All *Wnt* genes are expressed through to early larval stages (*L1*), prior to mouth opening. *Arrowheads* are color-coded to match schematics based on germ layer origins: *light blue*, ectoderm including tail fin (*fi*); *dark blue*, neural tube (*nt*) and cerebral vesicle (*cv*); *yellow*, endoderm derivatives including foregut (*fg*), hindgut (*hg*), preoral pit (*pp*), endostyle (*es*), club-shaped gland (*csg*), and gill slit primordium (*gs*); *orange*, mesendoderm; *red*, mesoderm derivatives including somites (*so*) and mesothelial cells (*me*); *fuchsia*, axial mesoderm derivatives including notochord (*no*); *white*, tailbud structures (*tb*, boxed), including neurenteric canal and chordoneural hinge; *black*, mouth primordium (*mo*). Note that only some somites are represented for the sake of clarity to permit visualization of underlying tissues. For all genes, anterior is to the left and dorsal up; developmental stages are indicated at the top and embryo schematics are shown at the bottom. Lateral and dorsal views are shown, with the exception of blastulae (*blast*), for which only lateral views are represented, and gastrula stage G3, for which lateral and blastopore (*white asterisk*) views are shown. *Dotted lines* indicate planes of sectioning in larvae L1. Scale bars = 50 μm. Please see Additional file 1: Text S1 for a detailed gene-by-gene description of all amphioxus *Wnt* expression patterns

**Fig. 3:**
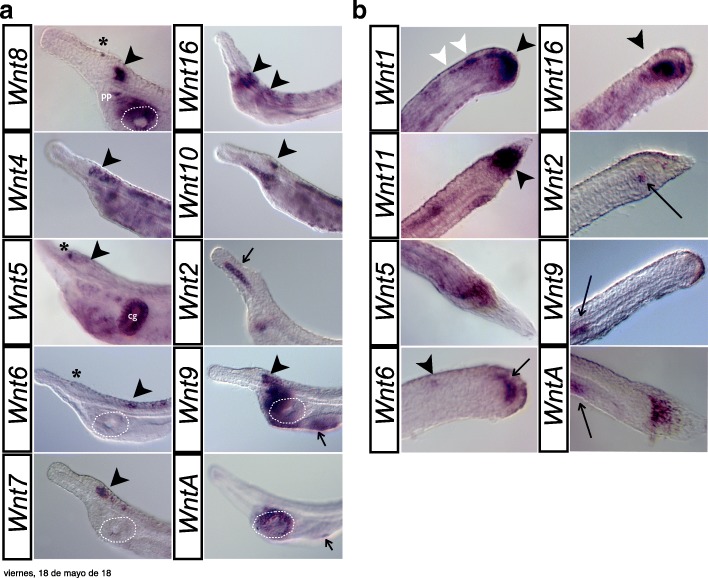
*Wnt* expression in one-gill-slit amphioxus larvae. **a** Anterior expression domains of selected *Wnt* genes ordered as in Fig. 2: *Wnt8*, posterior cerebral vesicle (*black arrowhead*), preoral pit (*pp*), endostyle, and around the mouth; *Wnt4*, posterior cerebral vesicle (*black arrowhead*) and neural tube; *Wnt5*, cerebral vesicle (*black arrowhead*), club-shaped gland (*cg*), and weakly in anterior notochord; *Wnt6*, isolated spots in neural tube (*black arrowhead*); *Wnt7*, posterior cerebral vesicle (*black arrowhead*); *Wnt16*, neural tube, including the hindbrain (*black arrowheads*); *Wnt10*, posterior cerebral vesicle (*black arrowhead*); *Wnt2*, anterior notochord (*black arrow*), and isolated cells of the anterior endoderm; *Wnt9*, posterior cerebral vesicle (*black arrowhead*), pharyngeal endoderm around the preoral pit, endostyle, and the first gill slit primordium (*black arrow*); *WntA*, around the mouth and in the first forming gill slit (*black arrow*) and within the rostral coelom and muscle. *Black asterisk* indicates pigment spot in the forming frontal eye; *white dotted line* indicates mouth. **b** Posterior expression domains. *Wnt1*, posterior neural tube (*white arrowheads*) and neurenteric canal hinge (*black arrowhead*); *Wnt11*, tail fin (*black arrowhead*); *Wnt5*, tailbud region; *Wnt6*, neural tube (*black arrow*) and posterior wall of the neurenteric canal (*black arrowhead*); *Wnt16*, posterior wall of the neurenteric canal and posterior mesoderm of the last somites (*black arrowhead*); *Wnt2*, posterior notochord (*black arrowhead*); *Wnt9*, midgut endoderm (*black arrowhead*); *WntA*, mesothelial cells (*black arrowhead*). Older larvae have tail fins containing brown pigment. All views are lateral, with anterior to the left and posterior to the right

**Fig. 4:**
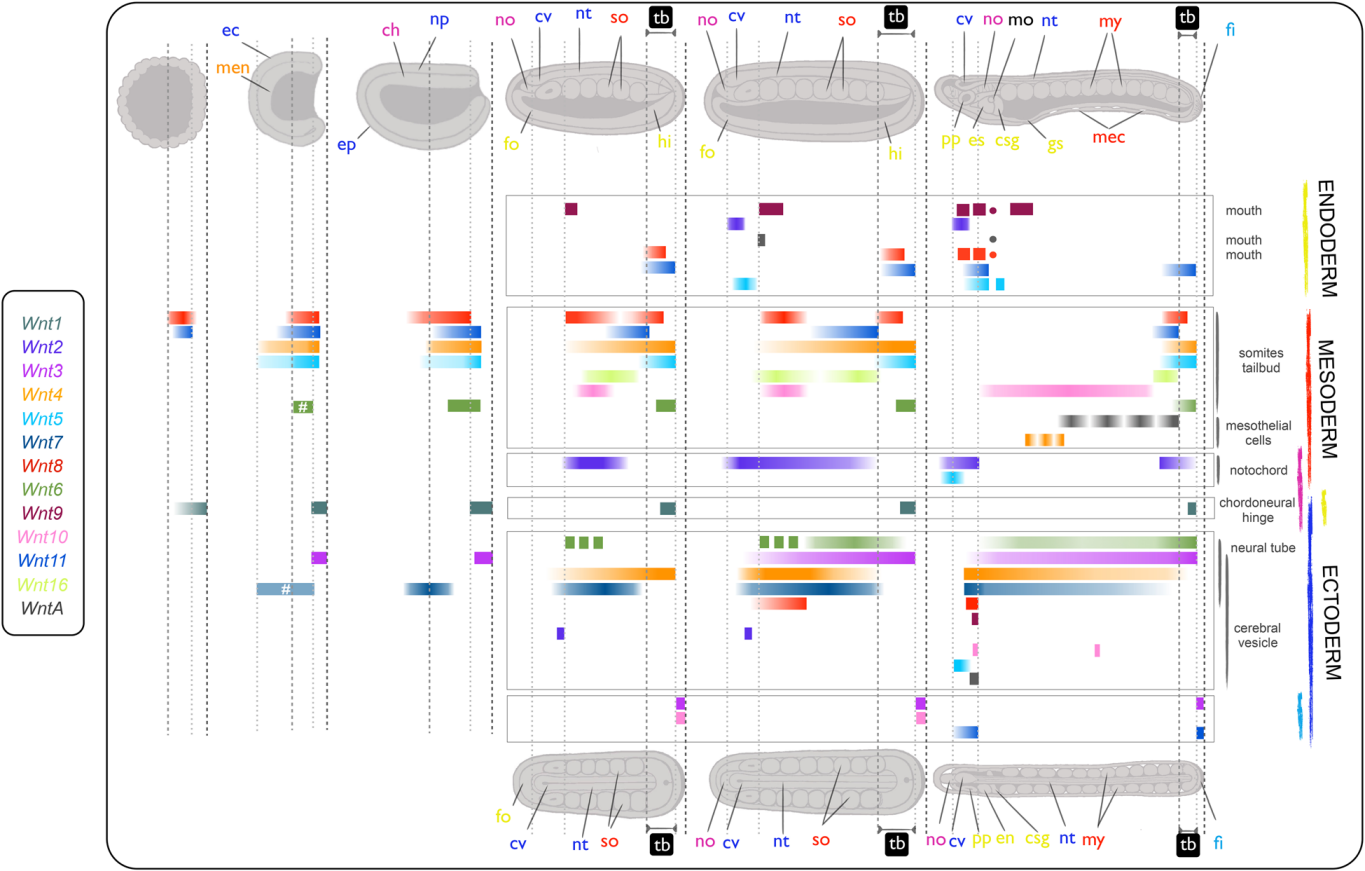
*Wnt* expression in *B. lanceolatum* is complex and spatio-temporally dynamic. Color-coded *horizontal bars* represent the expression domains of *Wnt* genes (see *left box*) and refer to schematic illustrations of *B. lanceolatum* embryos from blastula to early larva (lateral view at the *top* and dorsal view at the *bottom*; anterior is to the *left* and posterior to the *right*; main structures are labeled), organized by germ layer into endoderm (*yellow*), mesoderm (*red*), chordamesoderm (*magenta*), and ectoderm (*dark blue*, neural derivatives; *light blue*, epidermal derivatives). *Vertical dotted lines* delimit antero-posterior reference domains and underscore hypothesized Wnt functions during neural regionalization, posterior growth, and structure differentiation. The hash sign denotes minor differences with *B. floridae* expression. *Abbreviations*: *ec* ectoderm, *men* mesendoderm, *ep* epidermis, *ch* chordal plate, *np* neural plate, *no* notochord, *cv* cerebral vesicle, *fo* foregut, *nt* neural tube, *so* somites, *hi* hindgut, *tb* tailbud, *pp.* preoral pit, *es* endostyle, *csg* club-shaped gland, *gs* gill slit primordium, *my* myomere, *mec* mesothelial cells, *fi* fin, *mo* mouth primordium. See text for further details

The apparent conservative evolutionary stasis shown in amphioxus and the finding that amphioxus possesses a full and non-redundant Wnt repertoire make this organism a very attractive model to infer the roles of *Wnt* genes in the ancestral stem chordate, and from comparative studies, to analyze the impact of changes in the Wnt repertoire during the evolution of each chordate subphylum. In order to obtain the first comprehensive stage-matched developmental expression atlas for the cephalochordate Wnt repertoire, we performed whole-mount in situ hybridization (WMISH) for all *Wnt* genes in the European species *B. lanceolatum* (Figs. 2 and 3). Our results revealed that *Wnt* genes were expressed in a robust and precise tissue-specific fashion, with significant overlap among several amphioxus *Wnt* paralogs. Nevertheless, we also saw a number of differences in the expression of different paralogs, suggesting a choreographed modulation of their expression domains throughout development to generate spatio-temporally complementary patterns (a gene-by-gene detailed description of the expression patterns of all *Wnt* genes shown in Fig. 2 is provided in Additional file 1: Text S1 and summarized in Fig. 4). Overall, *Wnt* expression appears to be highly dynamic, spanning a broad variety of tissues derived from all three germ layers, which precisely up- or down-regulate different *Wnt* paralogs throughout development.

#### Posterior dominance of Wnt expression

“Posteriority” is likely the most conspicuous hallmark observed in the expression domains of the majority of amphioxus *Wnt* genes. At the blastula stage, *Wnt1*, *Wnt8*, and *Wnt11*, which were the first *Wnt* genes to be expressed in amphioxus (class A genes in Fig. 2), showed an evident restriction of their expression domains to the posterior half of the embryo. Thus, while *Wnt1* expression clearly labeled the vegetal pole, *Wnt8* and *Wnt11* were expressed at the equator of the prospective posterior pole [55]. At the gastrula stage G3, along with the early class A genes, *Wnt3*, *Wnt4*, and *Wnt5* expression domains appeared surrounding the blastopore (white asterisks, class B genes in Fig. 2), as well as *Wnt6* a little later (at stage G7). They were expressed in these posterior regions through neurulation (N2 and N3) until larval stages (L1). Close observation of the blastoporal view revealed some degree of overlap among gene expression patterns, but also important differences among the *Wnt* expression domains of each paralog. For instance, while *Wnt1* signal labeled the entire circumference surrounding the blastopore (Fig. 2, G3 column), *Wnt3* demarcated a narrower outer ectodermal strip of cells into G7 (Fig. 2). *Wnt8* and *Wnt11* signals were excluded from the edges of the blastopore and reached more central endomesodermal regions (Fig. 2). At G3, *Wnt4* signal was also strongly visible in the endomesoderm near the blastopore, but rather than being restricted to the posterior region, it spanned the entire layer surrounding the gastrocoel and was excluded entirely from the ectoderm (Fig. 2). Finally, *Wnt5* most strongly marked the endomesoderm of the dorsal blastopore lip (Fig. 2). Most of these early posterior *Wnt* expression domains were steadily maintained throughout development. The *Wnt1* expression domain, for instance, remained in the posterior wall of the neurenteric canal after elongation and closure of the blastopore until at least the early larval stage (Fig. 2, L1 column). *Wnt5* strongly marked the posterior growth zone during neurulation (Fig. 2, N2 and N3 columns), culminating in strong tailbud expression in larvae (Fig. 2, L1 column). *Wnt3* signal was observed up to L1 stage in the posterior-most ectoderm fated to become tailfin, abutting the *Wnt1* domain.

Posteriority was also observed for some *Wnt* genes with late expression onset (class C genes in Fig. 2). Of these, *Wnt10*, for instance, showed a new ectodermal expression domain in the most posterior part of the embryo at N2 and N3 (Fig. 2), which subsequently faded concomitantly with the appearance of *Wnt11* expression in this same region. This *Wnt11* expression clearly marked the entire fin in the one-gill-slit larval stage (Fig. 2, L1 column, and Fig. 3b). In summary, our data show that nine out of the 13 Wnt amphioxus *Wnt* genes showed posterior expression domains (Fig. 4), highlighting posteriority as one of the main hallmarks of this gene family.

#### Mesodermal Wnt expression and somite formation

In addition to the propensity for posterior dominance, another important source of *Wnt* signaling was observed in the presomitic and axial mesoderm, where seven out of the 13 *Wnt* paralogs were expressed. Besides the aforementioned early overlapping expression domains of *Wnt8* and *Wnt11* observed in the posterior endomesoderm surrounding the blastopore at G3, new expression domains of *Wnt4*, *Wnt5*, *Wnt6*, and *Wnt16* consecutively appeared in the most posterior mesoderm by G7, N2, N3, and L1, respectively, in a temporally orchestrated manner. At the one-gill-slit larval stage, *Wnt5* and *Wnt16* expression was maintained in the posterior-most mesoderm (Fig. 2, L1 column, and Fig. 3b). In addition to the *Wnt*-positive posterior mesodermal domain, several other *Wnt* expression domains could be identified in temporally dynamic complementary (as well as overlapping) patterns, in some cases forming nested domains along the anteroposterior axis (for instance, *Wnt8/16–Wnt11–Wnt8/16*–*Wnt4/5/6*). In other cases *Wnt* expression domains differed in their dorso-ventral extent within somites. For instance, *Wnt10* appeared excluded from dorsal domains compared with *Wnt16* in the N2 stage. *Wnt16* signal was observed in the last pair of formed somites by L1, while *Wnt10* appeared to be dorsally restricted in all mature somites (see cross-section in Fig. 2) and excluded from this last pair. No *Wnt* signal was observed in the anteriormost pair of somites at any stage of development.

In contrast to the paraxial mesoderm, surprisingly few *Wnt* genes appeared to be expressed in chordamesoderm or other mesoderm derivatives. For example, only *Wnt2* and *Wnt5* were expressed in the notochord. *Wnt2* signal was localized to the anterior two-thirds of the chordamesoderm during neurula stages (Fig. 2), becoming restricted to the most caudal and rostral portions in one-gill-slit larvae (Fig. 3); in contrast, *Wnt5* was conspicuously expressed in the anterior notochord only in larval stages (Fig. 2, L1 column, and Fig. 3). Finally, several non-myotomal structures of mesodermal origin appeared to express *Wnt* genes in restricted domains. *Wnt4* signal was observed in mesothelial cells in late neurula and early larval stages on the left side adjacent to the pharynx (Fig. 2). *WntA* was expressed in larval stages in mesothelial cells between the ventral endoderm of the gut and the ectoderm in both early and late larvae (Figs. 2, 3b, and 4).

#### Endodermal Wnt expression

In addition to mesodermal expression, *Wnt* signal was also evident in endodermal derivatives. By the gastrula stage, *Wnt8* and *Wnt11* expression domains were already observed in the endodermal portion delimiting the blastopore, expression domains that persisted until N3 and L1, respectively (Fig. 2). Many of the *Wnt* genes expressed in the posterior growth zone or tailbud also labeled hindgut domains in that area (e.g., *Wnt4*; Fig. 2). During larval stages, new anterior *Wnt* expression domains became evident in the anterior region and other parts of the digestive system (Fig. 2, L1 column). Some, such as *Wnt5*, *Wnt2*, and *Wnt9*, appeared to be already expressed at late neurula stages in anterior endoderm (Fig. 2, N2 and N3 columns). *Wnt* genes labeling specific derivatives by L1 included *Wnt8*, *Wnt9*, and *WntA* in the mouth primordium, *Wnt2*, *Wnt5*, *Wnt8*, and *Wnt11* in Haetschek’s diverticula, *Wnt8* and *Wnt9* in the endostyle, and *Wnt9* in the branchial primordium (Fig. 2, L1 column). Once the mouth was open and the first gill slit was clearly formed, *Wnt5* labeled the club-shaped gland, while *Wnt8* and *Wnt9* labeled the preoral pit, *WntA* the entire circumference of the mouth, and *Wnt9* and *WntA* the first gill slit (Figs. 2 and 3a). *Wnt4* and *Wnt2* signal appeared in a few cells of the endostyle along with *Wnt8* and *Wnt9*. *Wnt10* signal was also clearly evident in cells lining the rostral coelom (Fig. 3a). Posteriorly, *Wnt6* appeared to be expressed in a single line of cells demarcating the posterior wall of the neurenteric canal, and *Wnt9* labeled cells within the posterior gut (Fig. 3b).

#### Ectodermal Wnt expression

Ectodermal *Wnt* expression was detected in the epidermis as well as the nervous system. Besides the sequential coexpression of *Wnt3*, *Wnt10*, and *Wnt11* in the tip of the tail, the ventral epidermis of the anteroventral region at the level of the Haetschek’s diverticulum also appeared to express *Wnt11* and *Wnt5* in L1 larvae (Fig. 2, L1 column). At some stage, all *Wnt* genes—with the exception of *Wnt11*—labeled the developing central nervous system (CNS), consisting of cerebral vesicle and nerve cord (Figs. 3 and 4). Thus, the invaginating neural tube expressed *Wnt7*, *Wnt3*, *Wnt6*, *Wnt2*, *Wnt4*, and *Wnt8* starting in neurula stages, while *Wnt5*, *Wnt10*, and *WntA* signal appeared later in early larvae (Fig. 2). The spatio-temporal expression profiles differed among ligands, ranging from continuous *Wnt7* expression throughout the majority of the nervous system until larval stages, to the more restricted patterns shown by *Wnt2*, *Wnt4*, *Wnt6*, *WntA*, *Wnt10*, or *Wnt*5. Some, such as *WntA* or *Wnt10*, only labeled a few isolated cells within the cerebral vesicle in the early larval stage L1 (Fig. 2, L1 column). However, by the one-gill-slit larval stage, regionalisation of the cerebral vesicle became apparent, with *Wnt5* expression restricted to an anterior domain encompassing the frontal eye (Fig. 3a), while genes such as *Wnt4*, *Wnt7*, *Wnt8*, *Wnt9*, and *Wnt10* (and possibly *Wnt16*) marked posterior regions of the cerebral vesicle or the hindbrain (Fig. 3b).

### WntA expression in non-vertebrate chordates

We paid special attention to further investigating the function and evolution of our newly discovered chordate *WntA* genes. We therefore analyzed the expression patterns of *WntA* during embryonic development not only in the amphioxus *B. lanceolatum* but also in two additional chordate species, the ascidians *C. robusta* (Phelobobranchia order) and *H. roretzi* (Stolidobranchia order). *WntA* expression, however, was not detected during embryonic development in either of these two ascidian species (Additional file 1: Figure S2). This lack of expression of *WntA* was consistent with the absence of ESTs from embryonic libraries in databases of *C. robusta* and *H. roretzi*; the only existing ESTs of the *WntA* gene (FF969784 and FF969783 of *C. robusta*) came from adult animals. These results suggested, therefore, that *WntA* might be exclusively expressed at postembryonic stages or during adulthood in ascidians.

In contrast to ascidians, we found that *WntA* was expressed in a complex pattern during amphioxus embryonic development. Our results revealed that amphioxus *WntA* was the last *Wnt* gene to turn on in mid-late neurulae, with expression in only one or two cells located anteriorly on the left side under the ectoderm (Fig. 2), likely in the oral mesovesicle (OMV). The OMV is a coelomic vesicle that develops from the posterior ventral corner of the left first somite, and which has been associated with amphioxus mouth opening [56]. This expression domain persisted throughout development, accompanied by punctuated expression in the posterior cerebral vesicle from the late neurula until the pre-mouth larval stage (see above). *WntA* signal was also observed in cells between the ectoderm and endoderm of the forming gut of early and one-slit larvae (Fig. 2, cross-section, and Fig. 3), possibly in the mesothelial precursors of the “amoebocytes”, considered to be the homologs of the vertebrate blood cells [57]. Strong expression was further evident all around the mouth opening in these late larvae (Fig. 3a). This gene therefore represents a late-phase *Wnt* (class D) that plays a major post-neurulation role in differentiating structures such as the mouth and cerebral vesicle, and possibly in the circulatory system of amphioxus.

## Discussion

### Evolutionary patterns of Wnt subfamilies in non-vertebrate chordates

The identification and fully resolved phylogenetic reconstruction of the entire Wnt repertoire in several species of urochordate and cephalochordate (Fig. 1 and Additional file 1: Figure S4) permit the first complete reconstruction of the evolution of the Wnt subfamilies in chordates, revealing that each subphylum follows different evolutionary trajectories. Cephalochordates show a conservative evolutionary pattern, without either apparent gene duplications or gene losses since the cephalochordate lineage diverged from other chordates more than half a billion years ago [43, 50]. This finding suggests that the amphioxus genome has preserved the complete and prototypical chordate/deuterostome/eumetazoan Wnt repertoire (Fig. 1b). Analyses of many amphioxus gene families (e.g., *Hox* cluster [58], homeobox gene families [59], gene families of the steroid and retinoic acid signaling pathway [60–62], the tyrosine kinase superfamily [63], DNA-methylation genes [64, 65], and the FGF gene family [66]) reinforces the idea of evolutionary stasis of cephalochordate genomes [43, 44, 48]. The expression patterns of all *Wnt* genes analyzed here in *B. lanceolatum* are congruent with the expression of their eight orthologs previously characterized in the American lancelet species *B. floridae* [34–39] (*Wnt1*, *3*, *4*, *5*, *6*, *7*, *8*, and *11*; reviewed in [40]), which extends previous reports of cephalochordate stasis of morphology and developmental expression patterns [41, 46, 47, 67] and provides further support to the idea that the ancestral chordate resembled in many respects a modern amphioxus [58, 59].

In sharp contrast to the conservative nature of the amphioxus Wnt complement, ascidians show a dynamic pattern of evolution, including several gene losses and duplications (Fig. 1b). Some of the gene losses are likely ancestral, affecting the early stem of the ascidian lineage, since some paralogs are absent in all ascidian species. In contrast, other losses appear to be more recent, affecting only some groups of ascidians (i.e., *Wnt1* in the Phelobobranchia suborder and *Wnt11* in the *Molgula* genus), or limited to specific species (*Wnt3* to *B. schlosseri*). Inferring the point at which Wnt losses occurred is not only important to better understand their possible impact on the divergence of developmental mechanisms between ascidians and other chordates, but will also help elucidate how they may have contributed to genetic and morphological divergence during the ascidian radiation. Moreover, the extensive gene duplications of the *Wnt5* subfamily of the Stolidobranchia order may have also facilitated divergence within this order. Since this is the only case of amplification of all *Wnt* subfamilies analyzed in non-vertebrate chordates, and recurrent tandem duplications have independently affected several species of the Stolidobranchia, the developmental constraints to maintain *Wnt5* as single copy seem to have been exclusively relaxed in this order of ascidians.

**Fig. 5:**
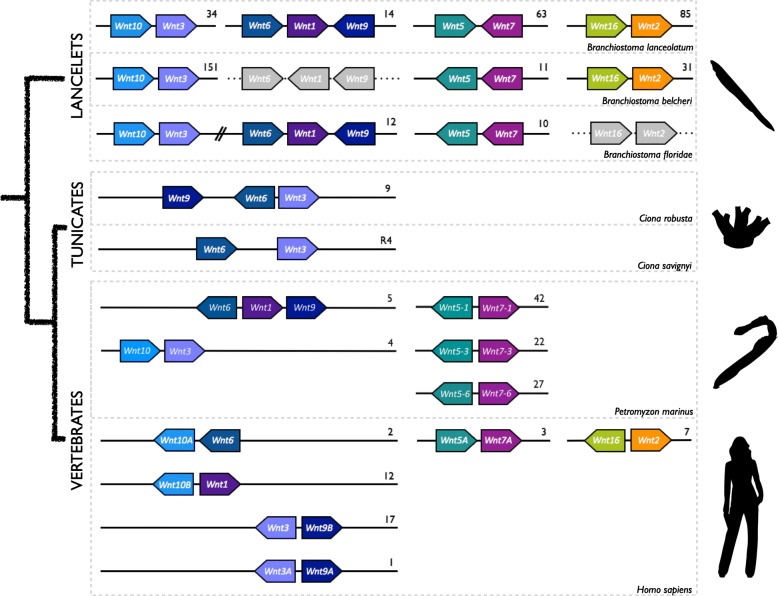
Synteny of *Wnt* genes in chordates. Genes and their relative position and orientation on chromosomes or scaffolds (*numbers above lines*) are represented by *block arrows*, color- coded by *Wnt* gene. *Gray arrows* on *dotted lines* indicate *Wnt* genes for which linkage has so far not been demonstrated. The phylogenetic relationship of chordate subphyla is shown on the *left*. Lancelets are represented by *B. floridae*, *B. belcheri*, and *B. lanceolatum*. Among tunicates, genome arrangements in *C. savigni* and *C. robusta* are shown. *P. marinus* and *H. sapiens* are vertebrate representatives. The data presented for *B. floridae* have been modified and extended from [25, 129]; this is the only cephalochordate for which *Wnt10–Wnt3* and *Wnt6–Wnt1–Wnt9* syntenic groups are linked on the same scaffold. Representative taxa are illustrated on the *right* (*black silhouettes*)

In contrast to cephalochordates and urochordates, many vertebrate Wnt subfamilies consist of two ohnologues (e.g., human *Wnt* subfamilies *2*, *3*, *5*, *7*, *8*, *9*, and *10*), which originated during the two rounds of whole-genome duplications (2R-WGD) that occurred during early vertebrate evolution [4]. The differences in the retention rate of *Wnt* paralogs between vertebrate and non-vertebrate chordates are possibly due to the different modes of duplication, i.e., genome duplication vs small-scale duplication (reviewed in [4, 8]), and suggest that duplication within Wnt subfamilies in non-vertebrate chordates might be impaired by dosage imbalance with the exception of the Wnt5 subfamily in the Stolidobranchia order. Remarkably, WntA is the only Wnt subfamily absent in vertebrates. To investigate when the *WntA* gene was lost, we analyzed the Wnt catalog of two additional vertebrate species: the lamprey *Petromyzon marinus*, an agnathan representative, and the shark *Callorhinchus milii*, a cartilaginous fish. We identified 24 Wnt sequences in *P. marinus* and 20 in *C. milii* databases (Additional file 1: Table S1). In our phylogenetic reconstruction, *P*. *marinus* and *C. milii* sequences distributed among all Wnt subfamilies, with the exception of the WntA subfamily (Additional file 1: Figure S4; see Additional file 3 for sequence alignment), suggesting that *WntA* was lost early in vertebrate evolution, before the divergence of jawless and gnathostome lineages. It can be argued, therefore, that *WntA* plausibly became dispensable in the primitive vertebrate either because alternative pathways or genes compensated for its function, or because environmental or physiological changes made them dispensable [8]. Finally, taking advantage of genomic information on the new *Wnt* genes identified in this study, we have re-evaluated the conserved synteny of previously postulated *Wnt* clusters [25]. Amphioxus has four clusters: the *Wnt9*–*Wnt1*–*Wnt6* cluster, the *Wnt2–Wnt16* cluster, the *Wnt3–Wnt10* cluster, and the *Wnt5–Wnt7* cluster (Fig. 5; Additional file 1: Table S3). In ascidians, we have found evidence for a single cluster: *Wnt9–Wnt6–Wnt3* in *C. robusta* and *Wnt6–Wnt3* in *C. savignyi* (Fig. 5; Additional file 1: Table S3). Lamprey conserves at least five clusters: the *Wnt9*–*Wnt1*–*Wnt6* cluster, the *Wnt3–Wnt10* and at least three *Wnt5–Wnt7* clusters (Fig. 5; Additional file 1: Table S3). Interestingly, cluster conservation is higher between lamprey and amphioxus than between either of these and human (*Wnt5–Wnt7*, and *Wnt5–Wnt7* and *Wnt2–Wnt16*, respectively), pointing to genomic rearrangements in the lineage leading to humans. In summary, our work highlights how distinct genomic rearrangements and patterns of gene conservation, loss, and duplication shaped differently the *Wnt* repertoire in amphioxus, ascidians, and vertebrates, and provides an evolutionary scenario that will facilitate future investigations of how these changes relate to adaptations to the environmental and physiological requirements evolved by each subphylum.

### Comparative analysis of Wnt expression patterns during embryonic development in the three chordate subphyla

Our assignment of all non-vertebrate chordate *Wnt* genes to the different Wnt subfamilies permits the first comparison of expression patterns of all orthologs among vertebrate and non-vertebrate chordates. With the complete cephalochordate dataset in hand, we were able to compare the expression patterns of all *B. lanceolatum Wnt* genes with the reported patterns of vertebrate *Wnt* genes and those available for ascidians (Additional file 1: Figure S3 and Text S2). Overall, our analysis revealed three main situations: first, cases of orthologous *Wnt* genes that share expression domains in homologous structures among chordate species, likely reflecting ancestral functional conservation; second, homologous structures that share *Wnt* expression domains, although the orthology of the ligands is not conserved among taxa, suggesting, therefore, gene function shuffling; and third, *Wnt* expression domains absent in amphioxus but present in other chordates, possibly reflecting lineage-specific *Wnt* innovations during the evolution of Olfactores (vertebrates + urochordates), or simplifications of the cephalochordate lineage.

#### Ancestral conserved Wnt functions in chordates

Amphioxus *Wnt7* is highly expressed in the CNS (Fig. 2), a feature that is shared with ascidian and vertebrate Wnt homologs [27, 34] (reviewed in Additional file 1: Figure S3 and Text S2). The posterior expression of *Wnt5* in somite and muscle development is also conserved in amphioxus (Fig. 2) ([39] and this work), vertebrates [68], and ascidians [28, 69]. *Wnt9* expression is conserved in endodermal derivatives in both amphioxus (Fig. 2) and vertebrates (specifically gut derivatives including the vertebrate liver), as well as in the gill slits, the vertebrate homologs of amphioxus pharyngeal arches, and in the amphioxus cerebral vesicle and the brain of vertebrates [70–73], while to our knowledge, only partial expression data have been documented for *Wnt9* orthologs in a colonial ascidian [33]. Finally, shared expression domains are observed for *Wnt10* and *Wnt16* in the neurectoderm, *Wnt16* in somites, and *Wnt5*, *Wnt3*, *Wnt8*, and possibly *Wnt11* in the tailbud (Additional file 1: Figure S3 and associated references). Globally, comparative analyses suggest a limited conservation in the expression patterns of orthologous Wnt subfamiles in the three chordate subphyla.

#### Function shuffling among Wnt paralogs

In vertebrates, expression of *Wnt1* is essential for proper anteriorposterior axial patterning of the brain and specification of particular neuronal populations (the “mid-hindbrain organizer”), in some cases functioning redundantly with other *Wnt* genes [74]. In amphioxus, no *Wnt1* expression has been observed in the anterior neuroectoderm or the cerebral vesicle ([38] and this study). However, we have identified a different ligand, *Wnt2*, with expression in the developing cerebral vesicle at mid-neurula stages, which is compatible with a role of amphioxus *Wnt2* in cerebral vesicle/hindbrain patterning. Other *Wnt* genes, including *Wnt3*, *Wnt5*, *Wnt7*, *Wnt8*, *Wnt9*, *WntA*, and *Wnt16*, are also turned on in the amphioxus brain between neurulation and larval stages (reviewed in [40] and this study). These results are consistent with the unexpectedly complex genoarchitecture in the amphioxus neural tube that is conserved with vertebrates [42] and highlights events of “function shuffling” [9] among Wnt paralogs during chordate evolution. Other remarkable examples of function shuffling can be found in the notochord, one of the defining synapomorphies of all chordates. Besides *Wnt5* and *Wnt11*, which are near-ubiquitously expressed and may play more general functions (perhaps in cell movement or polarity) across chordates, *Wnt2* is the only paralog expressed in the amphioxus notochord, while chick *Wnt16* (and maybe *Xenopus Wnt4* and *Wnt8* according to Xenbase data) is expressed in the vertebrate structure (Fig. 2; reviewed in Additional file 1: Figure S3). In contrast, *Wnt5* in *C. robusta* and the *Wnt-5α* paralog in *H. roretzi* are the only *Wnt* genes so far identified with expression in the asicidian notochord [26, 31, 75]. Similar examples of Wnt function shuffling are observed in early mesodermal derivatives (e.g., early paraxial mesoderm or somites), which express *Wnt10* in amphioxus (this work) but *Wnt3* in both vertebrates and ascidians (Fig. 2; reviewed in Additional file 1: Figure S3).

Comparison of the development of the posterior pole of the embryo in all three subphyla reveals a complex scenario in which a variable number of Wnt subfamilies take part in different species. While in ascidians, *Wnt5* seems to be the only ligand determining early posteriority in the primary axis, in amphioxus our study reveals a highly redundant posterior *Wnt* expression system involving at least eight out of the 13 subfamilies (i.e., *Wnt1*, *8*, *11*, and *3* surrounding the blastopore during gastrulation, plus *Wnt4*, *5*, *16*, and *6* in the most caudal part of the embryo later during neurulation and larval stages). In vertebrates, interestingly, while some species (similarly to ascidians) use a reduced number of Wnt ligands for determing early posteriority (e.g., *Wnt8a* in zebrafish [76], *Wnt11* (and *Wnt5*) in *Xenopus* [77, 78], and *Wnt3* in mouse [79] (reviewed in [11]), other species such as chicken show a redundant posterior Wnt system, more similarly to amphioxus (e.g., [80, 81]; Additional file 1: Figure S3 and associated references). If the ancestral chordate relied on a simple Wnt system for determing posteriority, extensive Wnt function shuffling occurred during the evolution of the cephalochordate lineage as well as some vertebrate species such as chicken, recruiting other Wnt subfamilies for this posterior signaling role. Evidence from the direct-developing hemichordate *Saccoglossus kowalevski* further corroborates the idea that posterior Wnt flexibility is a common occurrence during evolution, with a different but partially shared combination of *Wnt* genes showing blastoporal expression during gastrulation (*Wnt1*, *Wnt3*, *Wnt4*, *Wnt6*, and *Wnt16*) [82]. It seems, therefore, that providing β-catenin is asymmetrically localized (along the axis or in dividing daughter cells), then which particular Wnt ligands act upstream, or how they are spatially organized relative to one another, may not be particularly important [13], making the Wnt system one of the gene families most prone to function shuffling so far described. Importantly, the extensive events of function shuffling that occurred during Wnt evolution challenge the notion of establishing homologies simply based on the expression of orthologous genes and highlight the need to consider these events when analyzing cases of deep homology.

#### Wnt expression domains absent in amphioxus but present in other chordates

Different *Wnt* expression domains have been observed at different stages of germline formation and gonadogenesis in Olfactores, such as *Wnt5* in two ascidian species and mouse, *Wnt4* and *Wnt8* in zebrafish and mouse, *Wnt1* and *Wnt3* in chick, and *Wnt11* in *Xenopus* (Additional file 1: Figure S3 and associated references), while no evidence has been found suggesting any specific *Wnt* expression in cephalochordate primordial germ cells (PGCs) or the germline (this work and [46, 67, 83, 84]; reviewed in [49]). Similarly, the mesodermal cardiac-related expression domain of *Wnt9* in Olfactores—i.e., the heart endocardium of vertebrates [85] and the epithelial walls of the vasculature of the colonial ascidian *B.schlosseri* [33]—is not paralleled by any *Wnt* paralog in amphioxus. Moreover, in vertebrates, many *Wnt* genes are expressed in placodal derivatives and neural crest (Additional file 1: Figure S3 and associated references), and complex modulation of Wnt signals both within ectoderm and from other tissues is required for their specification and later differentiation [86–88]. In amphioxus, none of the ectodermal *Wnt* domains seem to be compatible with the presence of placode-like structures, which supports the notion that placodes may have been an evolutionary innovation of Olfactores [89–91], similar to migratory cells with neural crest-like properties [92]. It seems, therefore, that the appearance of important functional novelties during chordate diversification—e.g., germline, heart or placode development—were accompanied by new expression domains and functions of *Wnt* genes.

### Evolution of WntA: another new mouth?

Our work demonstrates the presence of *WntA* orthologs in both cephalochordates and urochordates, suggesting that its absence in vetebrates is likely due to a specific gene loss in this lineage. Our expression analyses reveal WntA function may be linked to mouth development in amphioxus, since it is clearly expressed in the region where the mouth will open at neurula stages, and around the periphery of the opening mouth in late larvae. A role for Wnt signaling in cephalochordate mouth formation may be further supported by the expression of Wnt antagonist *Dkk1*/*2*/*4* in the region in which the dissolution of basal laminae and mouth perforation occur [56].

After the dorso-ventral inversion of chordates (i.e., chordates are dorsoventrally inverted relative to non-chordates), the chordate mouth shifted from its now dorsal position [93], implying that chordates evolved a new means of mouth formation. Two possible evolutionary scenarios have been proposed for the origin of the amphioxus mouth, which would have had different consequences on the evolution of *WntA*. In the first scenario, the amphioxus mouth would share its evolutionary origins with the ambulacrarian coelomic pore-canal [56]. In this case, the absence of *WntA* expression associated with pore-canal formation in *Parentrotus lividus* [94] may suggest that WntA function was co-opted in the cephalochordate lineage (or secondarily lost in sea urchin). In the second scenario, the amphioxus mouth would represent a specialized gill slit [95] supported by the fact that both structures utilize Wnt (now including WntA), Fgf, and Hh signaling pathways for their formation [41, 96, 97], and that uncoupling of the gene regulatory network for mouth formation from the blastopore could be key deuterostome innovations. The expression of *WntA* in hemichordates is of particular relevance for discriminating between these hypotheses. Although evidence points to a conserved pharyngeal transcriptional network in deuterostomes [98], the expression patterns reported for *WntA* in *S. kowalveskii* are for stages too early to properly evaluate a putative conserved role in gill slit (or mouth) formation [82].

Regarding a role for *WntA* in ascidian mouth development, it should be noted that Olfactores and amphioxus primary mouths may not be homologous structures [56, 99]. To open a new mouth, Olfactores developed an anterior placode or stomodaeum, whereas amphioxus utilized an alternative system probably owing to its inability to form placodes [100]. With an alternative mode to open a mouth, Olfactores no longer needed *WntA* for this purpose, which might favor its loss in vertebrates and redeployment in urochordates. The dorsal-ventral inversion hypothesis, besides postulating changes in mouth formation mechanisms, proposes associated changes in brain architecture, which may have further relaxed constraints on *WntA* (and other *Wnt*) gene expression in this structure in different chordate lineages. Losses or redeployments of *WntA* have indeed been frequent during evolution. *WntA* has been lost in different species of arthropods, annelids, platyhelminthes and cnidarians [8], or used in a variety of conserved or novel structures and processes in different prototostome species [21, 101–104]. *WntA* evolution illustrates, therefore, the versatility of Wnt signaling in controlling diverse biological processes in metazoans.

### Functional diversification and loss of Wnt signaling during animal evolution

Function shuffling has significant consequences because it makes it difficult to predict biological function from orthology, challenging the so-called “orthology–function conjecture” (reviewed in [105]). This consequence is supported by the divergent expression patterns we observe here for orthologous chordate *Wnt* genes. Far from being a specific property of the chordate Wnt family, however, substantial differences have also been reported for *Wnt* expression in many other animal species, mainly arthopods and annelids [21, 25, 101, 106–110]. A detailed analysis of many *Wnt* genes in different protostome species, for instance, leads to the conclusion that the repertoire of Wnt ligands used during segment addition has evolved differentially among arthropod lineages, that the general role of *Wnt8* in regulating posterior development has been altered in annelids [21] and onychophorans [104], and that *Wnt5* and *Wnt16* orthologs are differentially expressed in annelids [25]. Outside bilaterians, substantial differences in the expression patterns of the ctenophore *Mnemiopsis leidyi Wnt* genes render difficult comparisons with those of other metazoans, including clades such as cnidarians, placozoans, and poriferans [111].

Finally, the loss of *WntA* in vertebrates, as well as of a number of *Wnt* genes in ascidians, illustrate another general feature of Wnt evolution: the “pervasiveness” of the loss of *Wnt* genes during animal evolution. The loss of Wnt subfamilies in ascidians might have contributed to the morphological diversification of urochordates. Likewise, the loss of *Wnt6* in hemipterans has been linked to the loss of maxillary palps in this group of insects [112], while the loss of *Wnt2* and *Wnt4* in insects [107] might be related with arthropod diversification. Thus, pervasive gene loss accompanied by numerous events of function shuffling appear to be two of the main features that characterize the evolution of the Wnt family not only in chordates but also in all branches of metazoan evolution.

## Conclusions

Up until now, Wnt research has mainly focused on identifying “conserved” biological functions. Here, we argue that essential information can also be gleaned from the analysis of Wnt “differences”, many of them derived from events of function shuffling and gene loss. Understanding the biological basis of such differences will help uncover how highly conserved developmental processes—such as axial patterning, germlayer specification, or body segmentation—might be controlled by molecular mechanisms (e.g., Wnt signaling) with remarkable genetic and functional diversity.

## Methods

### Genome database searches and phylogenetic analyses

Protein sequences of the Wnt repertoire from vertebrate *Homo sapiens*, urochordate *C. robusta*, and cephalochordate *B. floridae* were used as queries in BLASTp and tBLASTn searches in genome databases of selected species: http://amphiencode.github.io/ for *B. lanceolatum*; http://genome.bucm.edu.cn/lancelet/ for *B. belcheri*; https://blast.ncbi.nlm.nih.gov/Blast.cgi for *B. floridae*; NCBI Sequence Read Archive accession SRX437623 for *Asymmetron lucayanum*; http://www.aniseed.cnrs.fr/, https://blast.ncbi.nlm.nih.gov/Blast.cgi and http://octopus.obs-vlfr.fr/public/botryllus/blastbotryllus.php for ascidian species (*C. robusta*, *C. savignyi*, *P. fumigata*, *P. mammillata*, *H. roretzi*, *H. aurantium*, *B. schlosseri*, *M. occulta*, *M. oculata*, and *M. occidentalis*); https://genomes.stowers.org/organism/Petromyzon/marinus and https://www.ensembl.org/Petromyzon_marinus/Info/Index for *P. marinus*; and NCBI database for *C. milii*. Orthologies of the non-vertebrate chordate *Wnt* were initially assessed by the reciprocal best blast hit (RBBH) approach and corroborated by phylogenetic analyses. Phylogenetic reconstructions were based on ML inferences calculated with PhyML v3.0 and automatic mode of selection of substitution model [113] using protein alignments generated with MUSCLE [114] and CLUSTALX [115] programs and reviewed manually. Accession numbers and protein alignment for phylogenetic tree reconstruction are provided in Additional file 1: Table S1 and Additional file 2, respectively.

### Animal collection and gene expression analysis by WMISH

*C. robusta type A* adults were obtained from the National Bio-Resource Project for Ciona (AMED, Japan). *H. roretzi* adults were purchased from fishermen in Aomori and Iwate prefectures, Japan. *B. lanceolatum* adults were collected in Argelès-sur-Mer, France, and induced to spawn as in [116].

Fragments of *Wnt* genes were PCR amplified and cloned to synthesize gene-specific riboprobes for *H. roretzi* and *B. lanceolatum Wnt* genes (Additional file 1: Table S2). For *C. robusta*, cDNA clones were obtained from the cDNA clone collections [117, 118]. WMISH experiments were performed as previously described in [119] for *C. robusta*; as in [120] for *H. roretzi* with minor modifications (the acetylation step was not carried out before prehybridization, and after the antibody incubation the specimens were washed with PBST 12 times, 20 min each, and stored overnight at 4 °C); and as in [41] for *B. lanceolatum.* NBT/BCIP (Roche) or BMPurple (Roche) were used for the chromogenic reaction.

### Comparative studies of expression patterns

Vertebrate and ascidian *Wnt* gene expression patterns were identified and cross- and back- referenced using published literature as well as public database searches. These included ANISEED for ascidians species [121, 122] (http://www.aniseed.cnrs.fr/), ZFIN for zebrafish [123] (www.zfin.org), Xenbase for *Xenopus* [124–126] (http://www.xenbase.org/, RRID:SCR_003280), Geisha for chick [80] (www.geisha.arizona.edu/geisha/), and the EMAGE gene expression database for mouse [127] (http://www.emouseatlas.org/emage/). As no such database is currently available for cephalochordates, published literature images were examined by eye only. In all cases, special care was taken to ensure gene name nomenclature in the literature matched our results for *Wnt* gene assignation. In vertebrates, the expression of paralogs was grouped for ease of comparison across subphyla, under the assumption that both neo- and subfuctionalization events would be adequately represented. Please see Additional file 1: Figure S2 for additional details.

## Additional files

Additional file 1: **Figure S1.** Evolution of *Wnt5* in ascidians. **Figure S2.** Expression of *WntA* in two ascidian species. **Figure S3.** Chordate *Wnt* expression. **Figure S4.** Wnt subfamilies in *A. lucayanum*, *P. marinus*, and *C. milii*. **Table S1.** Chordate *Wnt* genes analyzed in this study. **Table S2.** *Branchiostoma lanceolatum* and *Halocynthia roretzi* primer and probe sequences. **Table S3**. *Wnt* synteny in lancelets (*B. lanceolatum*, *B. belcheri* and *B. floridae*) and vertebrates (*H. sapiens* and *P. marinus*).  **Text S1**.*Branchiostoma lanceolatum Wnt* expression as shown in Fig. 2. **Text S2.** References for Figure S3. (PDF 11566 kb)

Additional file 2: Wnt sequence alignment for Fig. 1. (FASTA 307 kb)

Additional file 3: Wnt sequence alignment for Additional file 1: Figure S4. (FASTA 99 kb)
