# Supplementary material for: Wnt evolution and function shuffling in liberal and conservative chordate genomes
Source: Genome Biol. 2018 Jul 25;19:98. doi: 10.1186/s13059-018-1468-3 (PMC6060547; doi:10.1186/s13059-018-1468-3)
Supplement: Supplementary file 1 — Figure S1. Evolution of Wnt5 in ascidians. Figure S2. Expression of WntA in two ascidian species. Figure S3. Chordate Wnt expression. Figure S4. Wnt subfamilies in A. lucayanum, P. marinus, and C. milii. Table S1. Chordate Wnt genes analyzed in this study. Table S2. Branchiostoma lanceolatum and Halocynthia roretzi primer and probe sequences. Table S3. Wnt synteny in lancelets (B. lanceolatum, B. belcheri and B. floridae) and vertebrates (H. sapiens and P. marinus). Text S1.Branchiostoma lanceolatum Wnt expression as shown in Fig. 2. Text S2. References for Figure S3. (PDF 11566 kb) [file 13059_2018_1468_MOESM1_ESM.pdf]

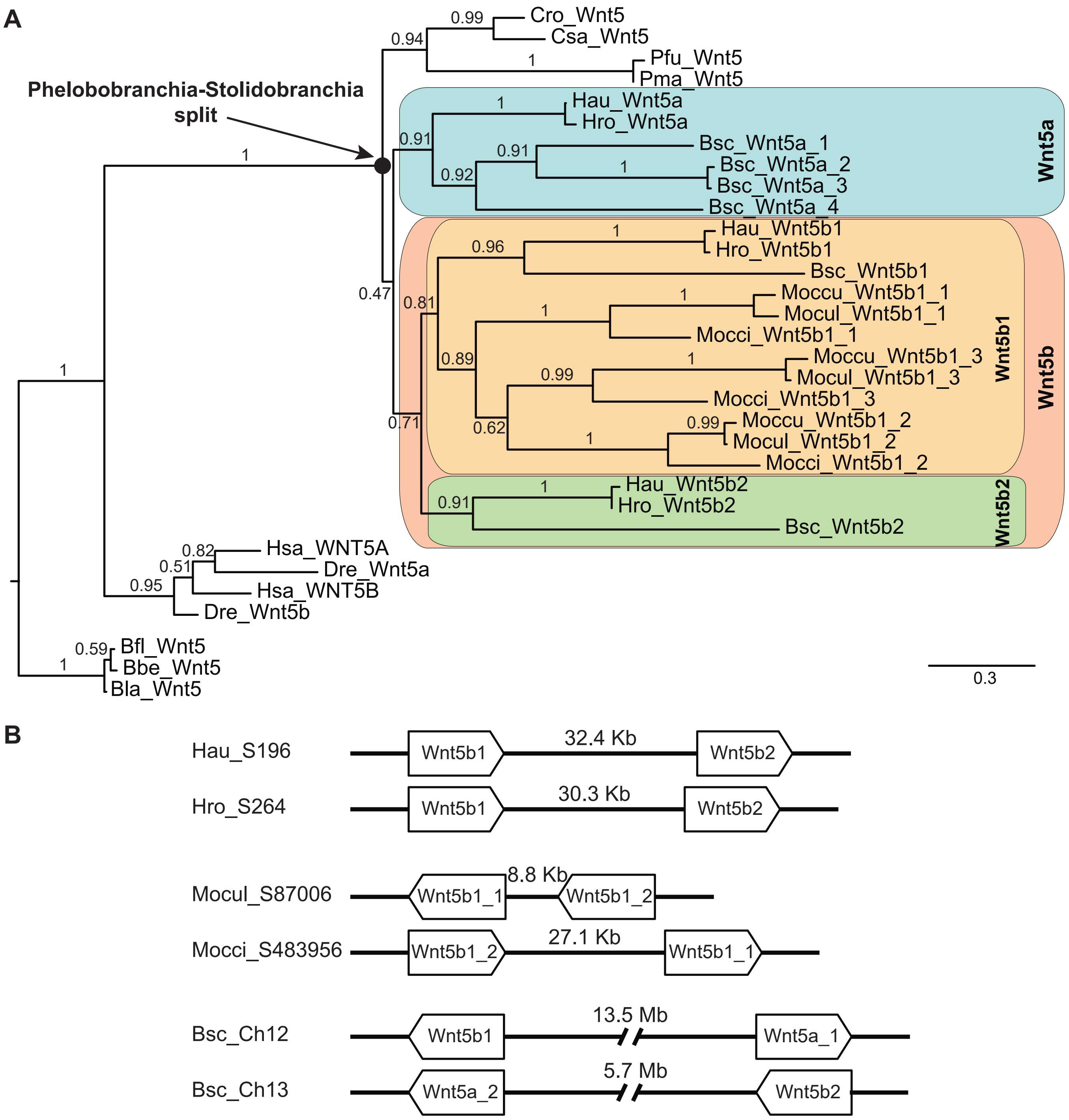

Figure S1. Evolution of *Wnt5* in ascidians. A. The ML phylogenetic tree of Wnt5 subfamily in ascidians suggests the presence of multiple paralogues (*Wnt5a* and *Wnt5b*) which originated in the Stolidobranchia clade after its split from Phlebobranchia. **B.** The fact that many of these *Wnt5* duplicates appeared to be located in the same genomic regions suggested that they originated by tandem gene duplications. Despite the fact that the number of *Wnt5* genes is the same in species of the *Molgula* and *Halocynthia* genera, the tree topology suggests that some of the duplications occurred after the splitting of the two genera (e.g. *Wnt5b1* and *Wnt5b2*), but before speciation within each group, in which further duplications independently occurred in different lineages (although ancestral *Wnt5* duplications in stem Stolidobranchia, followed by multiple gene losses and events of gene conversion in different species cannot be discarded). In any case, the expansion of *Wnt5* in Stolidobranchia provides a singular case of Wnt subfamily amplification in non-vertebrate chordates, suggesting that the evolution of this order of ascidian species has been accompanied by a relaxation of the evolutionary constraints that maintain *Wnt5* genes as single copy gene in other species. This may be linked to the recruitment of new *Wnt5* paralogues in biological innovations unique to this group of ascidians. Species abbreviations are as in Figure 1.

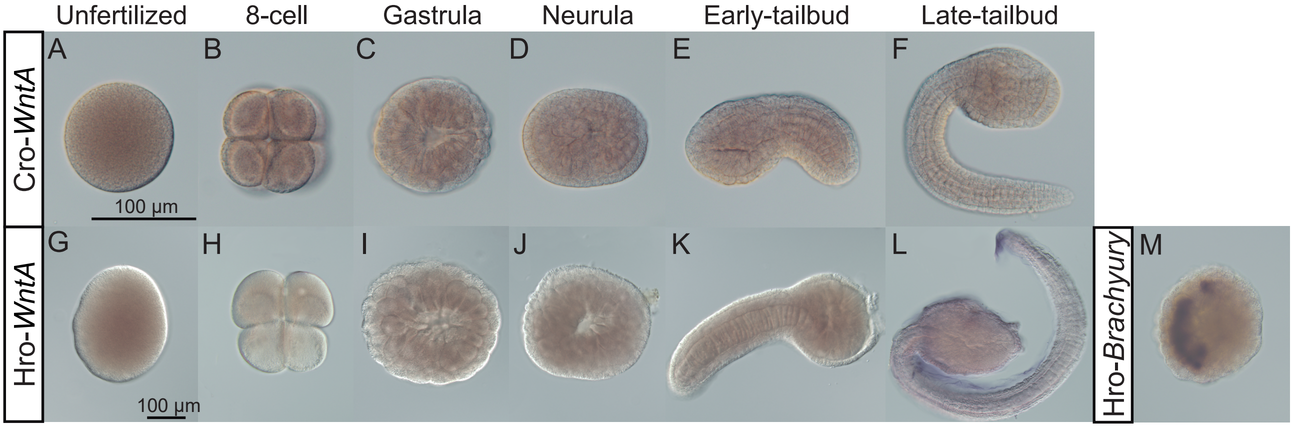

Figure S2. Expression of *WntA* in two ascidian species. Whole-mount *in situ* hybridization (WMISH) for *WntA* in *C. robusta* (Cro) (A-F) and *H. roretzi* (Hro) (G-L), and for the *Brachyury* gene in *H. roretzi* (M) as the positive WMISH control. No expression of *WntA* was observed in any selected stages –including unfertilized eggs, 8-cell, gastrula, neurula, early-tailbud and late-tailbud stages (a weak non-specific signal was observed in the tunic of *H. roretzi* late-tailbud larvae)–, whereas *Brachyury* expression was clearly detected in A-line and B-line cells (M). The lack of expression of *WntA* was consistent with the absence of ESTs from embryonic libraries in databases of *C. robusta* and *H. roretzi*.

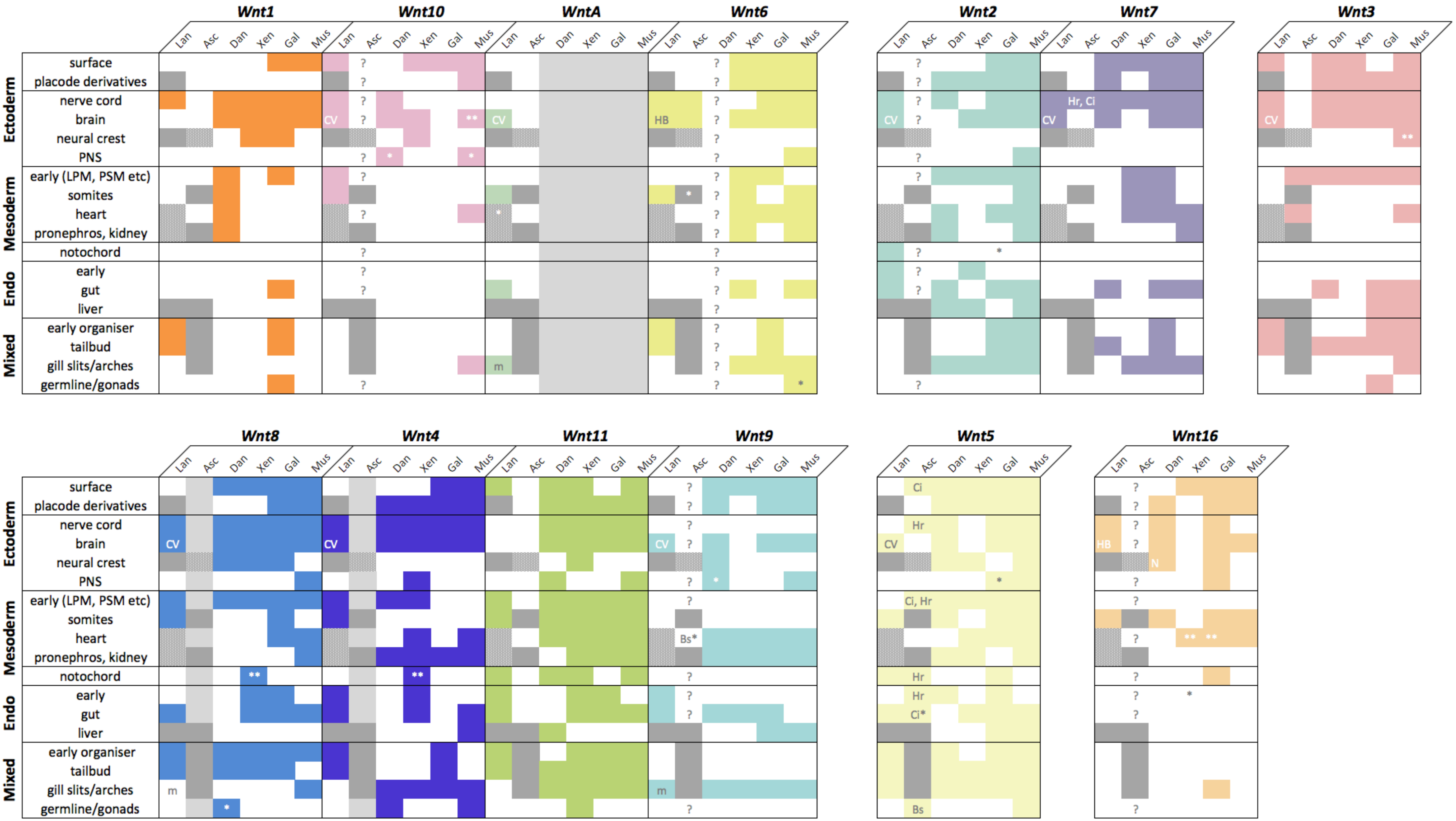
Wnt1
Wnt10
WntA
Wnt6
Wnt2
Wnt7
Wnt3
Wnt8
Wnt4
Wnt11
Wnt9
Wnt5
Wnt16
Lan
Asc
Dan
Xen
Gal
Mus
Ectoderm
Mesoderm
Endo
Mixed
surface
placode derivatives
nerve cord
brain
neural crest
PNS
early (LPM, PSM etc)
somites
heart
pronephros, kidney
notochord
early
gut
liver
early organiser
tailbud
gill slits/arches
germline/gonads
CV
HB
Hr, Ci
Ci
Hr
Ci, Hr
Ci*
Bs
Bs*
N
m

Figure S3. Chordate Wnt expression. Comparative expression for all Wnt subfamilies in chordates from data derived from our own results, published literature (see Additional file 1: Text S2 for references) and public databases (see Material and Methods section). Filled coloured boxes (Wnt subfamilies are coloured and grouped according to significant nodes in Fig. 1) denote documented expression; white filled boxes indicate absence of, or unreported expression; question marks are reserved for genes that are present, but whose expression has never been assessed; pale grey boxes represent lost genes (e. g. WntA in vertebrates or Wnt4 and Wnt8 in ascidians); dark grey boxes denote tissues/structures that are absent in the subphylum and/or at examined stages, and therefore, expression comparison is not possible (e.g. placode derivatives in amphioxus or somites in ascidians); stippled grey boxes highlight tissues/structures lacking clear homologues (e.g. heart in amphioxus or neural crest in ascidians). Expression patterns for vertebrate paralogues of the same Wnt subfamily were combined. Single asterisks (by gene order): *Wnt10* Dan: *Wnt10a* expression in neuromasts; *Wnt10* Mus: expression in ganglia; *WntA* Lan: a true heart does not exist but domains thought to be homologous have been identified with expression; *Wnt6* Asc: based on *WntE* orphan expression in muscle blocks (no somites present) in Imai et al (2004); *Wnt6* Mus: genital ridge; *Wnt2* Gal: convincing WMISH data in *Geisha* contradict a previous report of expression for which sequence ID could not be confirmed; *Wnt8* Dan: oocyte; *Wnt9* Asc (Bs): a true heart does not exist in colonial ascidians but expression in vasculature may reflect homology; *Wnt9* Dan: cranial ganglia; *Wnt5* Asc (Ci): endodermal strand; *Wnt5* Gal: ganglia; *Wnt16* Xen: hypochord. Double asterisks (by gene order): *Wnt10* Mus: various sources including knock-in expression reporter for *Wnt10a*; *Wnt3* Mus: indirect (loss of function); *Wnt8* Xen: *Xenbase* reports expression but this requires confirmation; *Wnt4* Xen: *Xenbase* reports expression but this requires confirmation; *Wnt16* Xen: suggested by Zhang et al (2011); *Wnt16* Gal: *Geisha* reports expression. Abbreviations: Lan=lancelet; Asc=ascidian; Dan=zebrafish *Danio rerio*; Xen=frog *Xenopus laevis/tropicalis*; Gal=chicken *Gallus gallus*; Mus=mouse *Mus musculus*; Ci=*Ciona intestinalis*; Hr=*Halocynthia roretzi*; Bs=*Botryllus schlosseri*; PNS (ectoderm)=peripheral nervous system; LPM/PSM (mesoderm)=lateral plate/presomitic mesoderm; CV=cerebral vesicle; HB=hindbrain; m=mouth.

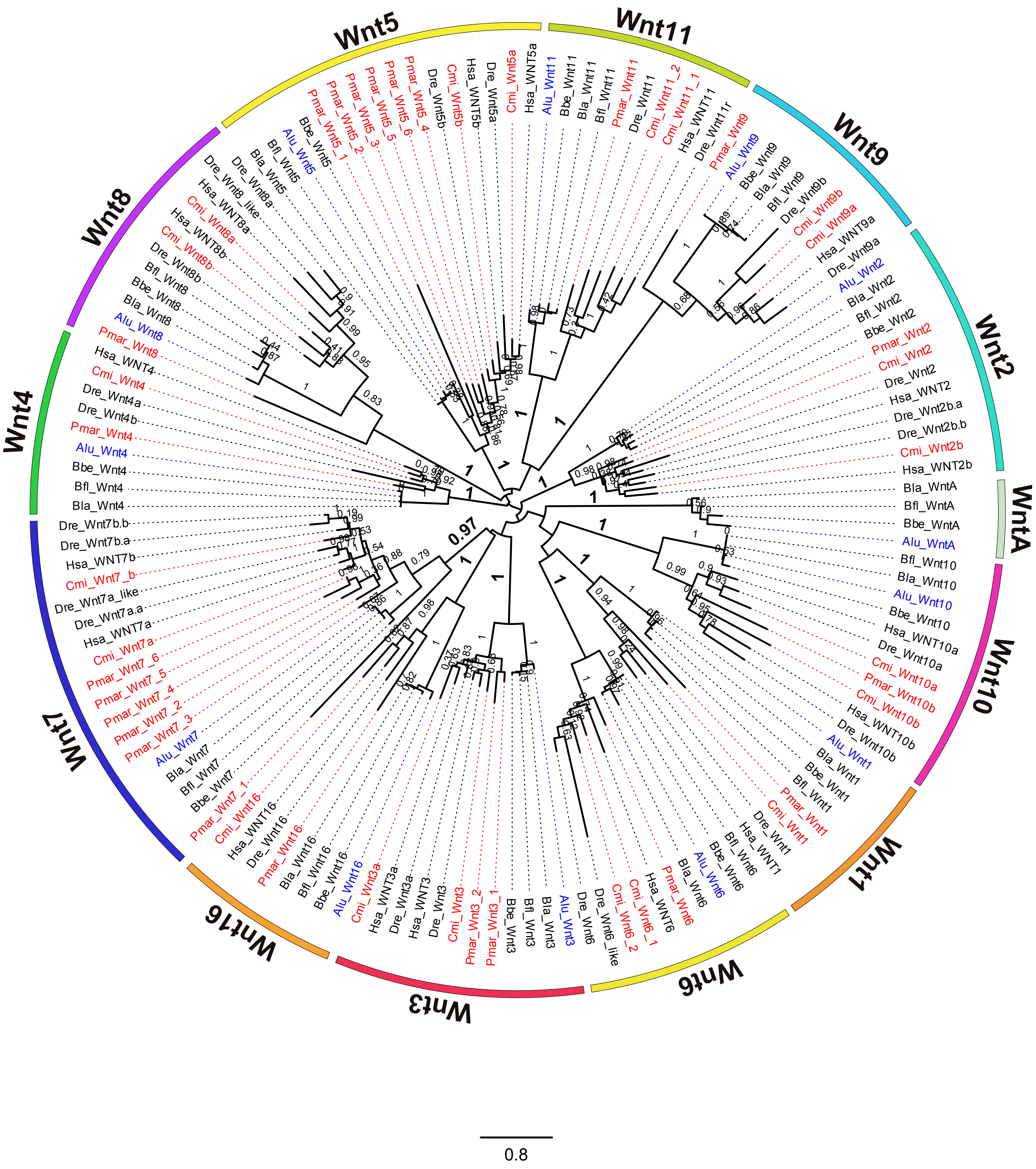

Wnt5
Wnt11
Wnt9
Wnt2
WntA
Wnt10
Wnt1
Wnt6
Wnt3
Wnt16
Wnt7
Wnt4
Wnt8
Pmar_Wnt5_1
Pmar_Wnt5_2
Pmar_Wnt5_3
Pmar_Wnt5_5
Pmar_Wnt5_6
Pmar_Wnt5_4
Dre_Wnt5b
Cmi_Wnt5b
Hsa_WNT5b
Dre_Wnt5a
Cmi_Wnt5a
Hsa_WNT5a
Bbe_Wnt5
Alu_Wnt5
Bfl_Wnt5
Bla_Wnt5
Alu_Wnt11
Bbe_Wnt11
Bla_Wnt11
Bfl_Wnt11
Pmar_Wnt11
Dre_Wnt11
Cmi_Wnt11_2
Cmi_Wnt11_1
Hsa_WNT11
Dre_Wnt11r
Pmar_Wnt9
Alu_Wnt9
Bbe_Wnt9
Bla_Wnt9
Bfl_Wnt9
Dre_Wnt9b
Cmi_Wnt9b
Cmi_Wnt9a
Hsa_WNT9A
Dre_Wnt9a
Alu_Wnt2
Bla_Wnt2
Bfl_Wnt2
Bbe_Wnt2
Pmar_Wnt2
Cmi_Wnt2
Dre_Wnt2
Hsa_WNT2
Dre_Wnt2b.a
Dre_Wnt2b.b
Cmi_Wnt2b
Hsa_WNT2b
Bla_WntA
Bfl_WntA
Bbe_WntA
Alu_WntA
Bfl_Wnt10
Bla_Wnt10
Alu_Wnt10
Bbe_Wnt10
Hsa_WNT10a
Dre_Wnt10a
Cmi_Wnt10a
Pmar_Wnt10b
Cmi_Wnt10b
Hsa_WNT10b
Dre_Wnt10b
Alu_Wnt1
Bla_Wnt1
Bbe_Wnt1
Bfl_Wnt1
Pmar_Wnt1
Cmi_Wnt1
Dre_Wnt1
Hsa_WNT1
Bbe_Wnt6
Bfl_Wnt6
Alu_Wnt6
Bla_Wnt6
Pmar_Wnt6
Hsa_WNT6
Cmi_Wnt6_1
Cmi_Wnt6_2
Dre_Wnt6_like
Dre_Wnt6
Alu_Wnt3
Bla_Wnt3
Bfl_Wnt3
Bbe_Wnt3
Pmar_Wnt3_1
Pmar_Wnt3_2
Cmi_Wnt3
Dre_Wnt3
Hsa_WNT3
Dre_Wnt3a
Hsa_WNT3a
Cmi_Wnt3a
Alu_Wnt16
Bbe_Wnt16
Bfl_Wnt16
Bla_Wnt16
Pmar_Wnt16
Dre_Wnt16
Hsa_WNT16
Cmi_Wnt16
Pmar_Wnt7_1
Bbe_Wnt7
Bfl_Wnt7
Bla_Wnt7
Alu_Wnt7
Pmar_Wnt7_3
Pmar_Wnt7_2
Pmar_Wnt7_4
Pmar_Wnt7_5
Pmar_Wnt7_6
Cmi_Wnt7a
Hsa_WNT7a
Dre_Wnt7a.a
Dre_Wnt7a_like
Cmi_Wnt7_b
Hsa_WNT7b
Dre_Wnt7b.a
Dre_Wnt7b.b
Bla_Wnt4
Bfl_Wnt4
Bbe_Wnt4
Alu_Wnt4
Pmar_Wnt4
Dre_Wnt4b
Dre_Wnt4a
Cmi_Wnt4
Hsa_WNT4
Pmar_Wnt8
Alu_Wnt8
Bla_Wnt8
Bbe_Wnt8
Bfl_Wnt8
Dre_Wnt8b
Cmi_Wnt8b
Hsa_WNT8b
Cmi_Wnt8a
Hsa_WNT8a
Dre_Wnt8_like
Dre_Wnt8a
0.8

Figure S4. Wnt subfamilies in *A. lucayanum, P. marinus* and *C. milii*. The ML phylogenetic tree reveals that each Wnt sequence from the cephalochordate *A. lucayanum* (in blue) grouped inside one of the 13 Wnt cephalochordate subfamilies, including the WntA subfamily, extending further the idea of genomic evolutionary stasis of cephalochordate species. In contrast, lamprey *P*. *marinus* and shark *C. milii* have Wnt representatives (in red) in all Wnt subfamilies except the WntA subfamily, suggesting that *WntA* was already lost before the divergence of jawless and gnathostome lineages. Notice that *Wnt5* and *Wnt7* have duplicated extensively in *P. marinus*. The scale bar indicates amino-acid substitutions. Values for the approximate likelihood-ratio test (aLRT) are shown at nodes. Species abbreviations: Cephalochordate species: *Asymmetron lucayanum* (*Alu*), *Branchiostoma belcheri* (*Bbe*), *Branchiostoma floridae* (*Bfl*), *Branchiostoma lanceolatum* (*Bla*); vertebrate species: *Petromyzon marinus* (*Pmar*), *Callorhinchus milii* (*Cmi*), *Danio rerio* (Dre), *Homo sapiens* (*Hsa*).

Table S1. Chordate *Wnt* genes analyzed in this study.

Subphylum	Gene	Previous names	Accession number*	ESTs/cDNAs*
**Cephalochordata**				
B. lanceolatum	*Wnt1*		BL08374	-
	Wnt2	*Wnt2b*[†]	BL18396	-
	Wnt3		ACE79725 (BL00565)	EU685300
	Wnt4		BL15100	-
	Wnt5	*Wnt5a*[†]	ACE79726 (BL05330)	EU685301
	Wnt6		ACE79727 (BL13401)	EU685302
	Wnt7	*Wnt7b*[†]	ACE79728 (BL12283)	EU685303
	Wnt8	*Wnt8a*[†]	BL09252	-
	Wnt9	*Wnt9a*[†]	BL22609	-
	Wnt10	*Wnt10a*[†]	BL23379	-
	Wnt11		BL18893	-
	Wnt16		BL18405	-
	WntA	*Wnt4*[†]	BL17431	-
B. floridae	*Wnt1*		AAC80432	FE564954, BW880884
	Wnt2		XP_002601759[‡]	-
	Wnt3		AAL37555	BW814403
	Wnt4		AAC80431	AF061973
	Wnt5		AAL37556	BW873866, FE573306, BW890381
	Wnt6		XP_002598625	-
	Wnt7	*Wnt7b* [1]	XP_002597288	BW953131, BW847525, BW859837, AF061975
	Wnt8		AAF80559	FE571808, FE547238, FE560742
	Wnt9	*Wnt14* [2]	XP_002598627	-
	Wnt10		XP_002598506 + XP_002598508 + EST FE577715.1[‡]	FE577715
	Wnt11		AAF80555	FE561860, FE553982, BW840509
	Wnt16		XP_002599799 + EST BW874763.1[‡]	BW874763
	WntA		XP_002609873	-
B. belcheri	*Wnt1*		Bb_306640F[‡]	HO762011
	Wnt2	*Wnt2b*[†]	Bb_073170F	JZ717686
	Wnt3	*Wnt3a*[†]	Bb_177200F[‡]	-
	Wnt4	-	Bb_271880F[‡]	-
	Wnt5	*Wnt5b*[†]	Bb_039350R+ Bb_039340R	-
	Wnt6	-	Bb_258720R	-
	Wnt7	*Wnt7b*[†]	Bb_039420R	-
	Wnt8	*Wnt8b*[†]	Bb_312060F	-
	Wnt9	*Wnt9a*[†]	Bb_185850F	-
	Wnt10	*Wnt10a*[†]	Bb_177150F	-
	Wnt11	*Wnt11b*[†]	Bb_034950F	-
	Wnt16	-	Bb_072770F	-
	WntA	*Wnt4*[†]	Bb_308600R	-
A. lucayanum	*Wnt1*		-	GETC01120369
	Wnt2		-	GETC01104091
	Wnt3		-	GETC01134136
	Wnt4		-	GETC01091176
	Wnt5		-	GESY01045804
	Wnt6		-	GETC01110690
	Wnt7		-	GETC01096614
	Wnt8		-	GETC01060203
	Wnt9		-	GETC01034191
	Wnt10		-	GETC01128999
	Wnt11		-	GETC01110192
	Wnt16		-	GETC01108796

	WntA		LZCU01110230‡	-
**Urochordata**				
Order Enterogona				
Family Cionidae				
C. robusta (formerly *C. intestinalis*)	*Wnt2*	*Wnt2* [3]<br>*Orphan Wnt-b* [4]	NP_001071794	BW191620<br>BW482592
	Wnt3	*Wnt3* [3, 4]<br>*Wnt3*; *Wnt10*†	XP_009859675	FF728330<br>BW498024
	Wnt5	*Wnt5* [3, 4]	NP_001027951	BW233701<br>BW479449
	Wnt6	*Orphan Wnt-e* [4]<br>*Wnt-b* [3], *Wnt11/1*†	NP_001071795	K048593<br>BW096149
	Wnt7	*Wnt7*† [3, 4]	XP_002128034	FF804473<br>BW311357
	Wnt9	*Wnt9/14/15* [3]<br>*Wnt14/15*† [4]	XP_009859770‡	BW504071
	Wnt10	*Wnt10/12* [3]<br>*Wnt10a/12* [4]	XP_002127850	FF855596<br>FF912944
	Wnt11	*Orphan Wnt-a* [4]<br>*Wnt-a* [3], *Wnt8 putative*†	NP_001028176‡	BP006149
	Wnt16	*Wnt-c* [3]<br>Orphan Wnt-d [4]	XP_002122330	-
	WntA	*Orphan Wnt-c* [4]<br>*Wnt-d* [3], *Wnt6 putative*†	XP_002121451	FF969783<br>FF969784
C. savignyi	*Wnt2*	-	Cisavi.CG.ENS81.R54.<br>2835750-2851101	-
	Wnt3	-	Cisavi.CG.ENS81.R4.<br>332380-338472‡	BW522473<br>BW552601
	Wnt5	-	Cisavi.CG.ENS81.R285.<br>233489-241411	BW573062<br>BW582532
	Wnt6	-	Cisavi.CG.ENS81.R4.<br>1192784-1201725	-
	Wnt7	-	Cisavi.CG.ENS81.R65.<br>368155-376728‡	BW591084
	Wnt9	-	Cisavi.CG.ENS81.R55.<br>213874-219426‡	-
	Wnt10	-	Cisavi.CG.ENS81.R926.<br>20876-28713	-
	Wnt11	-	Cisavi.CG.ENS81.R370.<br>21902-27494<br>Cisavi.CG.ENS81.R370.<br>21902-27525‡	-
	Wnt16	-	R48 (708195..713372)‡	-
	WntA	-	Cisavi.CG.ENS81.R19.<br>2776476-2790418‡	-
Family Ascidiidae				
P. mammilata	*Wnt2*	-	phmamm.CG.MTP2014.<br>S84.g02811	AHC0AAA87YF13_<br>RM1
	Wnt3	-	S291 (136784..144112)‡	-
	Wnt5	-	phmamm.CG.MTP2014.<br>S428.g08617‡	-
	Wnt6	-	phmamm.CG.MTP2014.<br>S211.g05449	-
	Wnt7	-	phmamm.CG.MTP2014.<br>S528.g09785‡	-
	Wnt9	-	S498 (49716..41376)‡	-
	Wnt10	-	S537 (95161..101893)‡	-
	Wnt11	-	S667 (23720..26900)‡	-
	Wnt16	-	phmamm.CG.MTP2014.<br>S12.g00518 + g00517‡	-
	WntA	-	S958 (26992..14509)‡	-
P. fumigata	*Wnt2*	-	S38291‡	-
	Wnt3	-	S37373 (311..151) +<br>S21003 (2454..478) +	-

			S21494 (914..1724)‡	
	Wnt5	-	S4452 (12623..3319)‡	-
	Wnt6	-	S1558 (2785..2204) + S12084 (4220..3294) + S1416 (453..4656)‡	-
	Wnt7	-	S553 (9591..1182)‡	-
	Wnt9	-	S2907 (11436..16644) + S4488 (12402..10747)‡	-
	Wnt10	-	S770 (23692..17150)‡	-
	Wnt11	-	S937 (16989..19608)‡	-
	Wnt16	-	S6225 (4783..3870) + S12301 (557..4227)‡	-
	WntA	-	S18 (28331..15021)‡	-
Order Stolidobranchia				
Family Pyuridae				
H. roretzi	*Wnt1*	-	Harore.CG.MTP2014. S634.g12371	DB583287 DB596795
	Wnt2	-	Harore.CG.MTP2014. S441.g09992	FY853847
	Wnt3	-	Harore.CG.MTP2014. S141.g00494‡	-
	Wnt5a	-	Harore.CG.MTP2014. S15.g14311	DB582570
	Wnt5b1	*HrWnt-5* [5]	Harore.CG.MTP2014. S264.g00180	FY863897 FY857344
	Wnt5b2	*HrWnt-5b* [6]	Harore.CG.MTP2014. S264.g00833	-
	Wnt6	-	Harore.CG.MTP2014. S139.g04292‡	-
	Wnt7	*HrWnt-7* [7]	Harore.CG.MTP2014. S12.g13523‡	-
	Wnt9	-	Harore.CG.MTP2014. S44.g02020‡	-
	Wnt10	-	Harore.CG.MTP2014. S118.g10881‡	-
	Wnt11	-	Harore.CG.MTP2014. S13.g11848	-
	Wnt16	-	Harore.CG.MTP2014. S355.g12638	-
	WntA	-	Harore.CG.MTP2014. S96.g13623‡	-
H. aurantium	*Wnt1*	-	Haaura.CG.MTP2014. S1095.g07586	-
	Wnt2	-	Haaura.CG.MTP2014. S270.g03873	-
	Wnt3	-	Haaura.CG.MTP2014. S461.g05183‡	-
	Wnt5a	-	Haaura.CG.MTP2014. S607.g05926	-
	Wnt5b1	-	Haaura.CG.MTP2014. S196.g03193	-
	Wnt5b2	-	Haaura.CG.MTP2014. S196_g03196	-
	Wnt6	-	Haaura.CG.MTP2014. S15.g00465‡	-
	Wnt7	-	Haaura.CG.MTP2014. S121.g02336‡	-
	Wnt9	-	Haaura.CG.MTP2014. S742.g06492‡	-
	Wnt10	-	S325 (666452..66801) + Haaura.CG.MTP2014. S426.g04986‡	-
	Wnt11	-	Haaura.CG.MTP2014.	-

			S64.g01495	
	Wnt16	-	Haaura.CG.MTP2014. S1551.g08535‡	-
	WntA	-	Haaura.CG.MTP2014. S1507.g08466‡	-
Family Molgulidae				
M. occulta	*Wnt1*	-	S716238 (22921..26525)‡	-
	Wnt2	-	S628454 (20043..26806)‡	-
	Wnt3	-	S655179 (2543..5965)‡	-
	Wnt5b1_1	-	S335521 (2809..8148)‡	-
	Wnt5b1_2	-	S211732 (585..2328)‡	-
	Wnt5b1_3	-	S400099 (9221..14111)‡	-
	Wnt6	-	S496787 (907..4017)‡	-
	Wnt7	-	S713940 (4242..7631)‡	-
	Wnt9	-	S379508 (8681..5606)‡	-
	Wnt10	-	S669497 (5717..2510)‡	-
	Wnt16	-	S321748 (11895..19107)‡	-
	WntA	-	S694937 (23190..26531)‡	-
M. oculata	*Wnt1*	-	Moocul.CG.ELv1_2. S108579.g10924	-
	Wnt2	-	Moocul.CG.ELv1_2. S29028.g01331	-
	Wnt3	-	Moocul.CG.ELv1_2. S127564.g14939	-
	Wnt5b1_1	-	Moocul.CG.ELv1_2. S87006.g06727	-
	Wnt5b1_2	-	Moocul.CG.ELv1_2. S87006.g06728‡	-
	Wnt5b1_3	-	Moocul.CG.ELv1_2. S109151.g11115	-
	Wnt6	-	Moocul.CG.ELv1_2. S129770.g15227	-
	Wnt7	-	Moocul.CG.ELv1_2. S117709.g13989	-
	Wnt9	-	Moocul.CG.ELv1_2. S127563.g14931 + g14932‡	-
	Wnt10	-	Moocul.CG.ELv1_2. S127563.g14924‡	-
	Wnt16	-	Moocul.CG.ELv1_2. S75012.g05236‡	-
	WntA	-	S113814 (30817..34341)‡	-
M. occidentalis	*Wnt1*	-	Moocci.CG.ELv1_2. S415796.g12520	-
	Wnt2	-	S643507 (63317..53607)‡	-
	Wnt3	-	Moocci.CG.ELv1_2. S649914.g30158+ g30159‡	-
	Wnt5b1_1	-	Moocci.CG.ELv1_2. S483956.g17278‡	-
	Wnt5b1_2	-	Moocci.CG.ELv1_2. S483956.g17276	-
	Wnt5b1_3	-	Moocci.CG.ELv1_2. S478739.g16781‡	-
	Wnt6	-	Moocci.CG.ELv1_2. S482528.g17137‡	-
	Wnt7	-	Moocci.CG.ELv1_2. S262076.g06136‡	-

	Wnt9	-	Moocci.CG.ELv1_2. S494455.g18504‡	-
	Wnt10	-	Moocci.CG.ELv1_2. S386552.g11160‡	-
	Wnt16	-	Moocci.CG.ELv1_2. S645945.g29523‡	-
	WntA	-	Moocci.CG.ELv1_2. S625464.g26812‡	-
Family Styelidae				
B. schlosseri	*Wnt1*	*wnt1* [8]	Boschl.CG.Botznik2013. chrUn.g54946‡	comp513900_c5_seq1<br>comp560097_c2_seq1
	Wnt2	*wnt2B* [9]<br>*wnt2b* [8]	Boschl.CG.Botznik2013. chr5.g27946‡	comp556459_c5_seq5
	Wnt5a_1	*wnt5A* [9]	chr12 (16990326..16997306)‡	comp564602_c4_seq4
	Wnt5a_2	-	Boschl.CG.Botznik2013. chr13.g54683‡	-
	Wnt5a_3	-	Boschl.CG.Botznik2013. chrUn.g52618‡	comp559388_c0_seq2
	Wnt5a_4	-	Boschl.CG.Botznik2013. chrUn.g69917‡	comp555181_c0_seq1
	Wnt5b1	-	Boschl.CG.Botznik2013. chr12.g59382‡	comp555286_c1_seq4
	Wnt5b2	*Wnt5b* [8]	Boschl.CG.Botznik2013. chr13.g39640 + g39642‡	comp560720_c1_seq3<br>comp560720_c1_seq2
	Wnt6	*Wnt2* [8]	Boschl.CG.Botznik2013. chr7.g45543 + Boschl.CG.Botznik2013. chrUn.g47754‡	comp542257_c0_seq<br>comp553141_c0_seq1
	Wnt7	*wnt7B* [9]<br>*wnt7a* [8]	Boschl.CG.Botznik2013. ChrUn.g46011‡	comp561241_c1_seq1
	Wnt9	*Wnt9B* [9]	chr12 (10811283..10817191)‡	comp566883_c1_seq16
	Wnt10	-	Boschl.CG.Botznik2013. chr6.g45564 + Boschl.CG.Botznik2013. chr6.g45565‡	comp547559_c0_seq1
	Wnt11	*Wnt4a* + *Wnt4* [8]	Boschl.CG.Botznik2013. ChrUn.g26693 + Boschl.CG.Botznik2013. ChrUn.g30622‡	comp555881_c2_seq1
	Wnt16	*Wnt16* [8]	Boschl.CG.Botznik2013. Chr9.g65512 + g65511‡	comp537474_c1_seq1<br>comp490118_c1_seq1<br>comp465456_c0_seq1
	WntA	*Wnt7b* [8]	chrUn (216959500.. 216964568) + Boschl.CG.Botznik2013. chrUn.g34723‡	comp557402_c0_seq1
**Vertebrata**				
P. marinus	*Wnt1*		scaf_00005‡: 20,134,359..20,157,546	ENSPMAT00000000452
	Wnt2		scaf_00042‡: 3,108,251-3,142,066	-
	Wnt3_1		scaf_00004‡: 12,290,358_12,311,172	ENSPMAT00000007828
	Wnt3_2		scaf_00028‡: 10,149,055..10,152,095	-
	Wnt4		scaf_00039‡: 6,074,861..6,105,243	ENSPMAT00000005487
	Wnt5_1		scaf_00042‡: 9,894,951..9,900,968	-
	Wnt5_2		scaf_05238‡: 2,865..8,297	-

	Wnt5_3		scaf_00022‡: 6,477,998..6,492,244	ENSPMAT00000003927
	Wnt5_4		scaf_00010‡: 2,668,595..2,676,578	-
	Wnt5_5		scaf_00068‡: 1,990,410..1,999,665	ENSPMAT00000010914
	Wnt5_6		scaf_00027‡: 8,182,445..8,189,929	-
	Wnt6		scaf_00005‡: 20,064,324..20,090,054	-
	Wnt7_1		scaf_00042‡: 6,386,092..6,420,339	-
	Wnt7_2		scaf_00057‡: 2,365,159..2,391,659	-
	Wnt7_3		scaf_00022‡: 7,178,423..7,194,222	-
	Wnt7_4		scaf_00049‡: 4,331,652..4,338,594	ENSPMAT00000001922
	Wnt7_5		scaf_00677‡: 7,202..37,080	-
	Wnt7_6		scaf_00027‡: 10,183,907..10,212,509	ENSPMAT00000006170
	Wnt8		scaf_00044‡: 1,493,431..1,505,124	ENSPMAT00000010173
	Wnt9		scaf_00005‡: 20,190,936..20,230,438	-
	Wnt10a		scaf_00004‡: 12,215,164..12,250,533	ENSPMAT00000007810
	Wnt11		scaf_00077‡: 1,067,696..1,071,303	-
	Wnt16		scaf_00038‡: 5,333,846..5,345,122	ENSPMAT00000009648
C. milii	*Wnt1*		XP_007907807	
	Wnt2		XP_007892784	
	Wnt2b		XP_007897604	
	Wnt3		XP_007905574	
	Wnt3a		XP_007884748	
	Wnt4		XP_007896096	
	Wnt5a		XP_007888715	
	Wnt5b		XP_007896697	
	Wnt6_1		XP_007906706	
	Wnt6_2		XP_007910098	
	Wnt7a		XP_007901542	
	Wnt7b		XP_007892915	
	Wnt8a		XP_007899559	
	Wnt8b		XP_007898078	
	Wnt9a		XP_007883762	
	Wnt9b		XP_007905575	
	Wnt10a		XP_007909548	
	Wnt10b		XP_007910099	
	Wnt11_1		XP_007909586	
	Wnt11_2		XP_007892018	
	Wnt16		XP_007903164	

*NCBI accession numbers are provided when available. Otherwise, accession numbers are from http://amphiencode.github.io/ for *B. lanceolatum*; http://genome.bucm.edu.cn/lancelet/ for *B. belcheri*; http://www.aniseed.cnrs.fr/ for ascidians; http://octopus.obs-vlfr.fr/public/botryllus/blast_botryllus.php for *B. schlosseri*'s ESTs database; https://genomes.stowers.org/organism/Petromyzon/marinus and https://www.ensembl.org/Petromyzon_marinus/Info/Index for *P. marinus*

†Name from the gene annotation in the database.

‡Sequences manually modified or annotated from the database.

References for Table S1

1. Schubert M, Holland LZ, Holland ND: Characterization of two amphioxus Wnt genes (AmphiWnt4 and AmphiWnt7b) with early expression in the developing central nervous system. *Dev Dyn* 2000, 217:205-215.
2. Putnam NH, Butts T, Ferrier DE, Furlong RF, Hellsten U, Kawashima T, Robinson-Rechavi M, Shoguchi E, Terry A, Yu JK, et al: The amphioxus genome and the evolution of the chordate karyotype. *Nature* 2008, 453:1064-1071.
3. Hotta K, Takahashi H, Ueno N, Gojobori T: A genome-wide survey of the genes for planar polarity signaling or convergent extension-related genes in Ciona intestinalis and phylogenetic comparisons of evolutionary conserved signaling components. *Gene* 2003, 317:165-185.
4. Hino K, Satou Y, Yagi K, Satoh N: A genomewide survey of developmentally relevant genes in Ciona intestinalis. VI. Genes for Wnt, TGFbeta, Hedgehog and JAK/STAT signaling pathways. *Dev Genes Evol* 2003, 213:264-272.
5. Sasakura Y, Ogasawara M, Makabe KW: HrWnt-5: A maternally expressed ascidian Wnt gene with posterior localization in early embryos. *International Journal Of Developmental Biology May* 1998, 42:573-579.
6. Miya T, Nishida H: Isolation of cDNA clones for mRNAs transcribed zygotically during cleavage in the ascidian, Halocynthia roretzi. *Dev Genes Evol* 2002, 212:30-37.
7. Sasakura Y, Makabe KW: Ascidian Wnt-7 gene is expressed exclusively in the tail neural tube of tailbud embryos. *Dev Genes Evol* 2000, 210:641-643.
8. Rosner A, Alfassi G, Moiseeva E, Paz G, Rabinowitz C, Lapidot Z, Douek J, Haim A, Rinkevich B: The involvement of three signal transduction pathways in botryllid ascidian astogeny, as revealed by expression patterns of representative genes. *Int J Dev Biol* 2014, 58:677-692.
9. Di Maio A, Setar L, Tiozzo S, De Tomaso AW: Wnt affects symmetry and morphogenesis during post-embryonic development in colonial chordates. *Evodevo* 2015, 6:17.

Table S2 ***Branchiostoma lanceolatum*** **and** ***Halocynthia roretzi*** **primer and probe sequences**

	Forward Sequence (5')	ReverseSequence (3')	Probe
Bla_Wnt1	GGAGAGAGTGCATGCTGTGA	TACAAGCACGTGTGGATGGT	ACTAGTGATTTACAAGCACGTGTGGATGGTCTTCGTCCTGACGCACTCCTC<br>GCACTTGACCTGACAGCACCACTGGAAGGTGCAGTTACACCGCTCTTTCGT<br>CACTTCCTGTCTAGTCGCGTAGCCTCGTCCGCAACACAGCAGGTCGCAGCC<br>ATCCAGCCCTCGTGACGTCACGTTGCACTCCCTCCCTCTTGTACCCTCGAA<br>GCCGAGACGGGGGTTGTTGCGACAGAAGTTGGGCGACCTCTCGTGGTAGAC<br>CAGATCGTTGTCCGTGGGGTACTTGTGGCGGGAGTTCTTGGGGACAAGCCC<br>GGTGACTTTCGCCCTGGAGCCCCGGTTGTTGCCGATGTCTGGCACCGCAAC<br>TTTAGACGCGCCGTCGAACTTCTCCTTCAAGCTGTCGCCGACGTCGCGGAA<br>GTTGGGCAGGCGCATCCAGCAGGTCTTGAGCGTGCAGGACCCCGACATGCC<br>GTGACACTTGCACTCTCTGCGTAGGTTCTCGGCTACAGCCACTCTGCCTGC<br>CTCGTTGTTGTGCATGTTGACCAGGTAGCGGACGGAGTCTCTCGTCTTCTC<br>TCCCGCATCCACGAACTGCTTGGCGAACTCCTTCCCGAACTCGACGTTGTC<br>GGAACATCCTCCCCATTCCCAGT
Bla_Wnt2	CGCACCATCTGTGACAACAT	GTCGATGATTTCGGAGCATT	ACTAGTGATTGTCGATGATTTCGGAGCATTCTTTACACCGGACGAAACAA<br>CACCAATGGAACTTGCACTCACACTTCCGTGACCTCTTCACCCTGGTGGTG<br>TCATAGCCCCGACCACAGCACATGATGTCGCAGCCATCAGTTCCGAGGGAC<br>GATCGGTTACATTGGCGACCCACCGTGCCGAGGGAACCTGTGTCATCATCG<br>GCTAAGCAGTAGTCTGGGGAGGTATCGAAGTACACCAGGTCTGACTTGGT<br>GGGTGTCTTGTGGTTCCTGTCGGACACGGTGAGTCTGCTGCCGCCCTGATT<br>CATCGTCACCTCAACCGCCCCATCATACCGCCGCCTCAGGTAGTCCGTCAC<br>TTTCCTGAAATGCGACATTGCCCTCCAGCAGGTGCGGAGAGTGCAGGACCC<br>GCTGACGCCATGACACTTACACTCCATCTTCATGTTCTTAGCGACGCCCCT<br>CCTCCCTGCCCTGTTGTTATGCATGTTCATGGCCGCTCGGGCGTCCCTCTG<br>GCTTCGGTCCCTCGCATCCACGAACTTCCTCGTGAAGCCCTCTCCGAACTT<br>GATGTTGTCGCTGCAGCCTCCCCACTCGAACTCACCCGTTGAGTCCTCGCT<br>GAATCCCCGCTTCTTGGGGTCACATG

Bla_Wnt3	AGTACTCCCGAGCGCGTTATG	TCACGTCATTTGCACGTGTGC	AGTACTCCCGAGCGCGTTATGGATTCAGTACGCTGCCAGGTGTCGGTCTTG<br>TTCCTGTTCGTCATTTGGTCTCAGACGGCGGACGTCGGCATGGGTTCTCCC<br>GGGTGGTGGTATCTGGCGGTGGGACCACAGTTCAGTTCACTGGCTGCTGCC<br>AATGCGGGAGGACAGGGGAGAAGATGGCCGCTGGTCTGCAGCAGTATACC<br>AGGTTTGGTGCCGAGACAGATCCGGTACTGCCGGAAGTTTCACGAGATCA<br>TGCCGTTCGTTGCCGACGGCACGAAGCTGGGCATCCGGGAGTGTCAGCATC<br>AGTTCCGCGGGCGCCGCTGGAACTGCACCACCGTCCAGGGACAGGTCTCCA<br>TCTTCGGACCAGTCTTAGACAGAGGAAACATCAGTACCACACCAAAAACAC<br>CTCCACGTGCGGGGGACTCCATACTCTGGCCGGCTGTCAACAGAGCTTCAA<br>GAGAAGCTGCCTTCGTACACGCCATCACCTCGGACTGTGGCTGCGACAACA<br>GACACAAGGGACCTCCTGGCGAGGGATGGAAGTGGGGAGGATGCAGTGAA<br>GATGTCTTCTTCGGCACCAAGTTCTCGCGAGACTTCGTGGACGCGAGAATA<br>CGGGGAAGGCGAGACGGGCGATCAGCCATGGACAGACACAACAATGAGGC<br>AGGCAGACAGTCTATCATGAAGAACCTGCAGCTGAAGTGTAAGTGCCACG<br>GCCTGTCGGGCAGCTGTGAGATCAAGACCTGCTGGTGGGCCCAGCCTGACT<br>TCCGCACGGTGGGAAACGTGCTGAAGGACAAGTACGACTCCGCCTCCGAGA<br>TGGCCGTGGAGCGCCACCGCGAGCCCTCCGGCATGGTCGACTCCCTCTACC<br>CGCGGTACAGCTTCTTCAAGGCGCCCGGTAAGGACGACCTCATCTACTTCG<br>AGAACTCGCCCAACTTCTGCGAGCCGAACAACTCCACGGGCTCGCTGGGAA<br>CCAGGGGGAGGGAGTGTAACATCACGAGCCACGGCATCGACGGGTGCCAGC<br>TGATGTGCTGCGGCAGGGGCTGGAACACGCGGACCGAGATGAGAACTGAG<br>AAGTGCCACTGCCAGTTCCACTGGTGCTGCTACGTCACGTGTCAGGAGTGC<br>CAGAAGAAGCACCAGGTGCACACGTGCAAATGACGTGA

Bla_Wnt4	TCTGCTACAGCGAACCAGTG	GTGCACCTCCACTGTCCTTC	ACTAGTGATTGTGCACCTCCACTGTCCTTCTACACGTTTTGCACTTGACAT<br>AGCAACACCAATGGAACTTGCAACTACATCTCTCCACTACTTCCCGGGTGT<br>GGGTGTTGTACCCTCTCCCGCAGCACAGAAGTTCGCAGCCATCGATGGCCT<br>TGGAAGTCTTGTTGCACACCCTCCCGACTGTCCCCATCGACCCCACCTTGG<br>TGTCCCGCACGCAAAAGTCTGGGGAAGCATCCAGATACACCAGGTCGGAGC<br>TCGAGTGCGGTTTGAAGTCAGAATTGAGCGGCACGAGTTCTCTCCTACTGC<br>CGATCTTTTTCTGTTGCACCTCGGTGGCGCCGTCGAATTTCTCCTTGAGCC<br>TCGCCCCGACTTCCCGGAACGGCGGCATGGCCCGCCAGCACGTCTTCAGCT<br>CGCAGGATCCCGAGACGCCGTGACACTTACACTCCGTCTTCATGTGGTCCA<br>CTAGGTTCCTCCTTCCGGCTTCATTGTTGTGCAGATCCATCAGGGCCCTGC<br>TCGAGGTCGCCGCGCCCGCTGCCGAGATGGAGTGCACGAAGGCAGCTTCCC<br>TCGTACCTTCTTCTAGGACCTTCCCAAACACAGGGCCTCGTCTGTTGATGA<br>GTGTGCTGCAGTTCCAGCGGCGGTGACGAAACTGGAACTGGCACTCTTCTA<br>TAGACATGCGGGCCCCCTCCTTTACACTGTCCATCACTT

Bla_Wnt5	TGATACCACGGTCTGAAGATG	AGTTGCGCCTCTGCTATTTGC	TGATACCACGGTCTGAAGATGGCGGTACAGATGAGTCTGCGGGTACTGCG<br>GGTGCTGGTGACACTGCTGTCCTGTTACACACACCTGGGCAGAGTCAGGGC<br>CACCTGGTGGCAAATGGCAGTGGATTCGAGATTGTACAGCCTGTCCCGGGC<br>GGAACTGTACATAATCGGGGCTCAGCCGCTCTGCACCTCGTTGGCGGGGCT<br>GTCGTCGGGACAGAGAAAACTGTGTAACTTGTATCAGGACCACATGGCGT<br>CTGTGGGCATCGGGGCCAGACAGGGGATAGAGGAGTGTCAGCACCAGTTT<br>AGGGACCGAAGATGGAACTGTACCACGTCGGACGAGGACTCCGTCTTCGGC<br>AGGATAGTCAACATAGGCAGTAGAGAAGCCTCCTTCACCTACGCCATAGCA<br>GCAGCAGGTGTAGTCAACGCCGTCAGTAGAGCATGCCGGGAAGGCGAGCT<br>GACCACATGCGGCTGCAGCAGAGCGAAGAGACCGAAGGACCTGAACAGGG<br>ACTGGCTGTGGGGAGGGTGTGGCGACGATGTGGAATATGGATATTACTTC<br>GCGCGAGAGTTTGTTGACGCTCAAGAGAAGGAGATCATCCCGTCGCCTGGG<br>TCCAGTGCGCACGCGCGGCAGCTGATGAACATGCACAACAATGAAGCTGGT<br>AGAAAGCTCACGTTCAGTAACGCGAGAGTAGCCTGCAAGTGCCACGGAGT<br>TTCCGGCTCCTGCAGCCTGAAGACGTGCTGGCAGCAGCTGGCGGACTTCAG<br>GACGGTCGGCAACCTGCTGAAGGACAAGTACGACGGCGCCAACGAGGTGA<br>AACTCATCCGGAGGGGCAAGAGGTACCGCCTGGATCGCCGCAACCCGAGAT<br>TCAACGTCTTCACGGACGAGGACCTTGTGTACCTGAATAAGTCACCCGACT<br>ACTGCAACGCGGACCCGACCATAGGTTCGCTGGGAACACACGGCAGAGAGT<br>GTAACAAAACCGGGCTGGGCACGGACGGGTGTAACCTCATGTGCTGCGGG<br>AGAGGATACAACACGTTCAAACGGGAAAAGGTGGAAAGGTGCAACTGCAA<br>GTTCCACTGGTGTTGTTACGTCAAATGCAAAAGGTGTCGGTCAATAGAAG<br>ACGTTTACGTATGCAAATAGCAGAGGCGGCAACT

Bla_Wnt6	CGGGGCTGCGACTACCAGAT	GAACGCCTCGCTCATAAACAC	CGGGGCTGCGACTACCAGATGAAGGGGGAGAGCCCGGATGGCAGCTGGGAGTGGGGGGGCTGCGGGGACGACATCGACTTCGGCTACACAAAGTCACGTGAGTTCATGGATGCCCAGACCAGACACAGGTCCGACATCAGAACGCTCCTGACTCTACACAACAACGAGGCGGGAAGACTGGCTGTAAAGAACTTTATGCGGACGGAGTGCAAGTGCCACGGACTGTCCGGATCATGCGCAGTAAAGACGTGTTGGAAAAAGATGCCGATATTCCGAGAGGTCGGGGTCCGGCTAAAGGAACGGTTCAACGGCGCGTTCCAAGTCATGGGATCCAACAACGGCAAATATCTCATCCCAGTCGGAGACACTATCAAAGCCCCTACGGCTGAGGACCTCGTCTATACCAACGAGTCGCCTAATTTTTGCAAAAGGAACAGAAAAACAGGGTCGCAAGGGACCAAAGGGCGGGCTTGTAACGCCACGTCCATGGGGATAGGCGGCTGTGACTTGTTGTGTTGTGGGAGAGGGTACAAGGAGAGACAAGTTGTCGTTGAGGAGAACTGCAAGTGTCGTTTCCACTGGTGCTGTGTAGTCAAGTGCTCCAAATGCACGGCCGTCAAAACTGTGCACGAGTGTTTATGAGCGAGGCGTTC
Bla_Wnt7	GAAGCTGCCTTTACTTACGC	GACGTCATGATCATTTGCAGG	GAAGCTGCCTTTACTTACGCCATCAGCTCGGCGGCGCTGGTGCACGCTATCGTCACAGCCTGCAGCCAGGGTAACATCTCTGACTGCGGCTGTGACCGTACGAAGGAGGGCGATCTGAACGACGAGGGCTGGAAGTGGGGCGGCTGCTCCGCCGACGTCAAGTACGGGCTTCGCTTCTGCAAGAAGTTCGTGGACGCCCGGGAGGTGGAGCAGAACGCGCGGGCTCTGATGAACCTCCACAACAATGAGGCTGGTAGGAAGGTGATTGACCAGCACACACGTCTGGAGTGTAAGTGTCACGGTGTCTCCGGCTCCTGCACCATGAAGACATGTTGGATTACTCTCCCACGCTTCCGAGAGGTCGGCAACATCCTGAAGGAGAAGTATCACCACGATTCCCAGCTCGTGGAAGCCGTTCGCGCCAGGCGCACGCGAAGGCCGACGTTTCTGAAACTGAAAAATTCTCGGACCTTCGAGAAACCTCGCGAGATTTCCCTTGTGTACCTGCGCGGGTCACCGAACTATTGTGAGCGTGACGAGGCAACAGGGTCGCTCGGAACGAACGGGCGGAGGTGCAACCGGACGAGCCCATATCAGGACGGGTGTGACCTGATGTGCTGCGGGAGGGGCTACAACACGCACCAGTTCGTCAAGACGTGGCAATGTAACTGTAAGTTCCACTGGTGCTGTTACGTCAAGTGCAACCAGTGCAGTGAGCGGACCGAGGAGTATACCTGCAAATGATCATGACGTC

Bla_Wnt8	CGATTCAAATGGGTGGACTT	GTGACTTCCGTGGTGATCCT	ACTAGTGATTCTGTTTCTTCTCCGCTGCTTTGACTTTTTCCGGACGTTTTT<br>CCTTCGGTTCTTGCTTTCCCGCTGCACACAGATGTATTTAGTGACGGTCTT<br>GGTGCACTGCGAACACTTGACCGAGCAACACCAGTGGAACTTGCAGTTACA<br>GCTGCTAGTGACTTCAGTCGTGATCCTTTTCGGAACGTATCCGCAGTCCTT<br>ACAAAGGCGCTTGCAACTCTTCTTCTCGTATTTGTCCATGTTTTTCCCTCC<br>GCGGAGGCATTCACGTCCAAGAGTTCCCCGTGACCCTACGGTGAGGTTCTG<br>CCTACAGTAGTCGGGGGAATCCTCAAGGAACACCATGTCCTTCTTAAGCCC<br>GGTGTTCCTCTTGGAGCTGGCGCTGTTGTTCTCCGTCAGCTGACCGCGCAC<br>GTAGTCCACCTTGTCCGCCTTCTTGTACTTCTTCTTGAGGAAGACGCCGAT<br>GGCGCGGAAGTCGGCCAGCTGGAGCCAGCATGTCTTGGTGGTGCAGCTTCC<br>CGACACGCCGTGACACTTACACACCCGCTTCATGGTCTGTCTCACCGCCTT<br>CCTGCCCACATCGTTGTTGTGTAGGTTCATGGCTGCCCGGGCGTCCTGGCC<br>GTTCTCAACCCCGTCCGAGTACCTCTTGGAGATGCTCTCTCCGAAGGAGAT<br>GTCGTCACTACAGCCGCCCCAAGTCCAACCGCCTTCCGCTTTTTTCCCGTT<br>GTTGGTCTGGTCGCACCCGCATTGCTCGAAAGCGCCCTTGCTGCGGTTCCT<br>GGTCAGGACGTACATCACACCAGCGGCGCTTATGGCGTGCACGAA

Bla_Wnt9	GGAGTGGACGACATCAACG	TCTCGTGTTGCATTCTTTGC	GACAAGCTGGAGCTCACCGCGGTGGCGGCCGCTCTAGAACTAGTGGATCCC<br>CCGGGCTGCAGGAATTCACTAGTGATTTCTCGTGTTGCATTCTTTGCTCTC<br>GTGTTGCATTCTTTGCTCTCGTGTTGCATTCTTTGCACTCGACGTAACAAC<br>ACCATTTGACCTGGCACTGGCAAGGTCTCTTGATGATCAGGCTCTGTGTGT<br>TGTGCCCCCTCCCGCAGCAGATGCTGTCGCAGTTCTTTTCCTTGTCGCAGA<br>TGCGCCCTCTTGTCCCGTATGAATATTTCCCCTTTTTGCAGTAGCTCGGAG<br>ACTCGTCGACGAACACCATGTCGTTGTTCTTCGGGCCTTTAGGCGCCTCGT<br>TCCCGACGGGCTCGTTCATGAGCCGCGCTCGCTCCCCCGCACCGTTGGTCA<br>TGCTGACAACCTTCATGGCCTTCTCATACTTGTTTTTCAAGACGTCTCCGA<br>TCTCGTGGAAGGGTGCGAGTTGTCTCCAGCACGTCTGGACGCTGCAAGACC<br>CCGAGACGCCATGACACTTACACGTCGTCTTCACCCGGGACCGCACCACTC<br>GGATTCCCAGGTTGGTGTTATGTCTGTCCACGGCGGCTCTCAGGTCCTGCT<br>CCTCCGCCTTGTTCATACTGAGGAACTGGCGCGCGAATTTCCGAGAATATT<br>TCAGGTTGTCCCCGCACCCTCCCCACAGCCACGTCTCGCGGTTGTCGATGT<br>CGTTAAAACTCTCGTCGCACGTGCACCGCTCCAGCTTCCCGCTCGAGCACG<br>CCCGGCCCAGGCGTGGGTCAGCCCGGAGGACGAGAT
Bla_Wnt10	AAGATTCCGGAAGACCCTGT	ATTCTGTGATTCGGCACTCC	ACTAGTGATTAAGATTCCGGAAGACCCTGTGCTGAACGCCAACACCGTGTG<br>CCGCACTTTCCCCGGGCTCAGCAAGAAGCAGCTCCAACTGTGTTACGAGTA<br>TCCCGACGTGACGGCGGCGGCCATACAGGGCGTGCAGATCGCCATTCACGA<br>GTGTCAGTGGCAGTTCAAGAACCACCGATGGAACTGTTCCAACCTGGAGAC<br>CAAGAACAAGAACCCGCACCTCACACAGATCGCTAGCAAAGGTATCCGAGA<br>GACAGCCTTCACCTTTGCGTCGGTATCCGCGGGAGTGGCTCACGCTGTGGC<br>CAACGCCTGTAGCCTGGGTAAGCTGCACACGTGCGGCTGTGACAATGACTA<br>CCCCACCAAGCCTCAGATCCCTACCTACTCCCCCAGCTCATTCCCAGGGGCC<br>ATCCCTGGCGGCCCGATCCCAGGCTACCCTCCCGGGTACACACAGGGACAG<br>ACGGCGGTACTACACACGAACAGTGGCGGCGGCAAGTCGTACAACGATTGG<br>TCGTGGGGAGGTTGTAGCCACAACATTGAGTACGGCATCAAGTTTTCCAA<br>GGATTTCCTTGACTCTCGCGAGACGTCGGTGGACATCTTCTCG

Bla_Wnt11	TTGCCGATGTAGGGAAGAAC	ACCGTGAAGTCGTTGTAGCC	ACTAGTGATTCCCACAGCACATTGTTTGACAGCTGTCTGGACCAACTGACG<br>TCTTGTTGCACAACCTTCCTGTAGTTCCGTACGACCCCTTCCTGTTGTTAA<br>CTATACAGTAGTCTGGAGACTTGTCCACGAAGATCAGATCCCCGCTGCTGT<br>GTCGGCGGGACCGTCTGTCCTCTGGGACTAGCTGTTGTCGTGTTCCGATCT<br>TCCTCTTCACCATCTTGATAGCGTATGAATATTTCTCGGCGAGTTCGTGAG<br>AGATCTCTGTCAGGTCGGCTAACGACTTCCAGCATGTCTTGACGTTACATG<br>ATCCCGAGACGCCATGACACTTGCACTTCGTCGTCATCGTGTTTTGCACGG<br>CCAGTCTTCCAGCTCCACTGTTATGTAGGTTCATGAGTGTCTGTGTGTGAG<br>ACCGCTTCCTCTTCATCATGGGTGCGTCCGCGAACCCGGTACCGAACTCCA<br>GCCCGAACTTGACGTTGTCCCCACAGCCGCCCCACGTGTAGTTCCCGTCAG<br>GCTTCTCGCCGGGTTTCCTCGCGCATGAGCAGGCCTT
Bla_Wnt16	ATGGACGGGGCTAATAAAGC	ATGATGGTCTCGCAGGTGTT	ACTAGTGATTATGATGGTCTCGCAGGTGTTGCACTTGACGTAACAGCACCA<br>GACGAACTTGCAGCCGCCACCTCTCCACGAACCGAACCACCTGCGTGTTGT<br>AGCCCCGCCCGCAGCACAGCAGGTTACAACTGTCCGGACCCGTGGACGTTC<br>TGTTACACTCTCTTCCCCTGGTGCCAAAGATCCCACGTTTCTTGTCCGACT<br>TGCAGAAGTTTGGAGATTTCTCAAGAAACACCAACTCCTCGTCTCCGATAG<br>GTACCTTCCGGTGCATCTTCTGTTTCCGCCGTAACCGCCGTCTCGTCTTCC<br>GGAGGATGGGGACTGACGTCATGTACTTCTTTTTCAACATATCACCAACCC<br>GTTTGAAGCTGGGCAATGTTTTCCAACAGGTCTTTACTGCGCATGATCCGG<br>ACACGCCGTGACACCTGCACTTG

Bla_WntA	AACGCCATCAGGTGGTCTAC	TACAAGTGACCTCGCAGCAC	ACTAGTGATTTACAAGTGACCTCGCAGCACCATTTGAATTTGCAGTTGCA<br>ACTCTCTTCTGTGGTCTCCTCTCTCGACACGAAGTCTCTGTTACAACAGAG<br>TAGCTGACAGCCGTCTACCCCCGCACTTGTTCTATTACATTTCCGCCCGCG<br>CGTCCCGTACGACCCCTTGGCCACGTCGACTTCGCAGTAGTCGGGAGAATC<br>GTCCAGATACACCACGTCGTGTGCTGTGGGGCGTTTGTGTCTTTTGTTTCT<br>CGGACGTAATCTGGATCCCTTTTTGTTGATCTTGACGTACGTGGCTCCGTG<br>GTACTTTCCCATGATAATGTCTCCGACCTGTCGAAAGCTCGGCATGGACTC<br>CCAACAGACCATGGTGGCGCAAGACCCGGACACGCCGTGGCACTTGCAGGT<br>AACCTTCATATTGGCCTTGATTGACTTTCTGCCTGCTTCGTTGTTGTGTAA<br>GTTCATTAGTCCAGTATCGTCTGCGGCCACTTCATTAGCGTCCATAAACTC<br>TTTGGACAGGTTGTCTCCGAACCTGATGTTATGGGAGCACCCTCCCCAATC<br>CCAC

Hro_WntA	GTACCGGCCCCGAACTCC	TCACTTGCAATATGATTCTTCAAC	GTACCGGCCCCGAACTCCGACCAACAAACACGAAATTCAAAAGGAGCGCGA<br>GCCTACTGCAATTCACTAAAATACCTCACAGATAAGCAGAAGAGTTTGTG<br>CATCCACAGTCCCACGATAATGAGTAAAATTAGCCACGGTGTTATCGCTGG<br>AATGAGAGAATGTCAATTGCAATTTCAACACAGGAGATGGAACTGTACAC<br>CGTATCAATCAAATAATCCGAAGACCCATGCTTTCGAACCTGTGCTGAAAA<br>ACAAGGGCCCTGAAAGAGCGTACATCAAAGCCATGCTGAGCGCAGCCGTGT<br>CCTATAAGATAACGCGAGCGTGTTCTGGCGGTGAACTTCCTCTTGAATGCA<br>TGTGTGCAAATATTAAACGTCCAGCGAAGGATGATCCGGAGGACCCTAAG<br>AAAAAATTTGAATGGGGAGGATGCTCGGATGATGTTAATTTTGGAGATTC<br>GAGATCCCGTGAATTCTTTGACGAATCGGATTTATCTCGAAATCAAGGAA<br>GAGTACTTGACATACACAACAATGAAGCGGGAAGAAAGTCCGTAAAAATG<br>AGCAATCGTACAACATGCAAATGCCACGGCCTTACCGGAGCCTGCACACAG<br>AACATTTGCTGGAGATCGCTACCTCCATTGCGCAGAATAGGTAAAGAATT<br>ATTTCAACGATATAATAGCGCTGTACGAGTAAAAACTCGAAAACTGGATA<br>TCCAAGGAAAGGCTACAACCAGCGATTTGGTATACGTTCGAAAATCACCT<br>TCGTTTTGCATAAGAAACAATCGTGGTTCTTACGGGACCGCCAAACGGTG<br>GTGTAACAGGACAAGCACGGAAACTGAGGGATGCGCTCATATGTGCTGCG<br>GTCGGGGTTACAGAACCGAAGAAAAGATAAAGATAACTGAATGCAATTGC<br>GAATTTCAATGGTGTTGTAATCTCAATTGTTATTTCTGCGAAGAACATGT<br>TGACGAATCATATTGCAAGTGA

Table S3. ***Wnt*** **synteny in lancelets (*B. lanceolatum, B. belcheri* and *B. floridae*) and vertebrates (*H. sapiens* and *P. marinus*)**

LANCELETS

	Branchiostoma lanceolatum#		*Branchiostoma belcheri*		*Branchiostoma floridae**	
Gene	ID	Scaffold: coordinates (strand)	ID	Scaffold: coordinates (strand)	ID	Scaffold: coordinates (strand)
Wnt1[¶]	BL08374	Sc0000014: 2,655,007-2,664,370 (-)	306640F	Sc0001290: 14,548-21,893 (+)	113720	sc0000012: 3,339,473-3,348,336 (+)
Wnt2	BL18396	Sc0000085: 1,065,381-1,083,862 (+)	073170F	Sc0000031: 837,724-858,936 (+)	145693	sc0000061: 1,676,862-1,683,402 (+)
Wnt3	BL00565	Sc0000034: 1,039,202-1,073,486 (-)	177200F	Sc0000151: 348,528-368,321 (+)	118309	sc0000012: 804,098-834,674 (+)
Wnt4	BL15100	Sc0000105: 395,585-434,158 (-)	271880F	Sc0000613: 51,484-51,936 (+)	56711	sc0000323: 231,613-265,780 (+)
Wnt5	BL05330	Sc0000129: 22,035-62,251 (+)	039340R	Sc0000011: 585,989-598,190 (-)	118160	sc0000010: 3,427,102-3,453,255 (-)
Wnt6	BL13401	Sc0000014: 2,678,147-2,708,947 (-)	258720R	Sc0000490: 126,712-138,581 (-)	57222	sc0000012: 3,308,009-3,322,220 (+)
Wnt7	BL12283	Sc0000266: 95,574-120,318 (-)	039420R	Sc0000011: 721,380-731,165 (-)	56634	sc0000010: 3,594,285-3,614,777 (-)
Wnt8	BL09252	Sc0000095: 692,325-710,019 (+)	197740F	Sc0000194: 438,989-439,780 (+)	56726	sc0000413 :614,838-634,936 (-)
Wnt9	BL22609	Sc0000014: 2,622,645-2,644,219 (+)	185850F	Sc0000168: 197,245-210,888 (+)	67024	sc0000012: 3,362,909-3,377,125 (-)
Wnt10	BL23379	Sc0000034: 1,116,089-1,170,047 (-)	177150F	Sc0000151: 250,803-287,391 (+)	113709	sc0000012: 753,835-754,254 (+)
Wnt11	BL18893	Sc00000711: 287,178-311,798 (-)	034950F	Sc0000009: 654,648-663,318 (+)	271417	sc0000460: 536,632-547,782 (+)
Wnt16	BL18405	Sc0000085: 387,208-427,572 (+)	072770F	Sc0000031: 137,433-139,798 (+)	205854	sc0000027: 1,292,590-1,325,871 (+)
WntA	BL17431	Sc0000101: 315,161-333,725 (+)	308600R	Sc0001374: 7,955-16,915 (-)	60204	sc0000201: 663,106-672,419 (-)

VERTEBRATES

	Homo sapiens
Gene	Chromosome: coordinates (strand)
Wnt1	12: 48,978,453-48,981,676 (+)
Wnt2	7: 117,276,631-117,323,289 (-)
Wnt2b/Wnt13	1: 112,466,541-112,530,165 (+)
Wnt3a	1: 228,007,051-228,061,260 (+)
Wnt3	17: 46,762,506-46,833,154 (-)
Wnt4	1: 22,117,305-22,143,969 (-)
Wnt4b	
Wnt5a	3: 55,465,715-55,490,539 (-)
Wnt5b	12: 1,529,891-1,647,243 (+)
Wnt6	2: 218,859,821-218,874,233 (+)
Wnt7a	3: 13,816,258-13,880,121 (-)
Wnt7b	22: 45,920,362-45,977,129 (-)
Wnt8a	5: 138,083,892-138,092,365 (+)
Wnt8b	10: 100,463,041-100,483,744 (+)
Wnt9a/Wnt14	1: 227,918,656-227,947,898 (-)
Wnt9b/Wnt15	17: 46,833,201-46,886,730 (+)
Wnt10a	2: 218,880,363-218,899,581(+)
Wnt10b/Wnt12	12: 48,965,340-48,971,763 (-)
Wnt11	11: 76,186,325-76,210,736 (-)
Wnt11r	
Wnt16	7:121,329,003-121,341,104 (+)

	Petromyzon marinus
Gene	Scaffold: coordinates (strand)
Wnt1	scaf_00005: 20,134,359-20,157,546 (+)
Wnt2	scaf_00042: 3,108,251-3,142,066 (+)
Wnt3_1	scaf_00004: 12,290,358-12,311,172 (+)
Wnt3_2	scaf_00028: 10,149,055-10,152,095 (-)
Wnt4	scaf_00039: 6,074,861-6,105,243 (-)
Wnt5_1	scaf_00042: 9,894,951-9,900,968 (+)
Wnt5_2	scaf_05238: 2,865-8,297 (-)
Wnt5_3	scaf_00022: 6,477,998-6,492,244 (-)
Wnt5_4	scaf_00010: 2,668,595-2,676,578 (+)
Wnt5_5	scaf_00068: 1,990,410-1,999,665 (+)
Wnt5_6	scaf_00027: 8,182,445-8,189,929 (-)
Wnt6	scaf_00005: 20,064,324-20,090,054 (-)
Wnt7_1	scaf_00042: 6,386,092-6,420,339 (-)
Wnt7_2	scaf_00057: 2,365,159-2,391,659 (+)
Wnt7_3	scaf_00022:7,178,423-7,194,222 (+)
Wnt7_4	scaf_00049: 4,331,652-4,338,594 (+)
Wnt7_5	scaf_00677: 7,202-37,080 (+)
Wnt7_6	scaf_00027: 10,183,907-10,212,509 (+)
Wnt8	scaf_00044: 1,493,431-1,505,124 (-)
Wnt9	scaf_00005: 20,190,936-20,230,438 (+)
Wnt10	scaf_00004: 12,215,164-12,250,533 (+)
Wnt11	scaf_00077: 1,067,696-1,071,303 (+)
Wnt16	scaf_00038: 5,333,846-5,345,122 (-)

# scaffolds 129 and 266 are in fact linked (new scaffold 63), demonstrated through additional PacBio sequencing (Robinson-Rechavi, personal communication)
* alternate scaffolds exist in many cases due to presence of two alleles during genome sequencing, and may explain opposite orientations
[¶] Shared colour denotes *Wnt* genes on the same chromosome or scaffold

Text S1. *Branchiostoma lanceolatum Wnt* expression as shown in Figure 2.

The amphioxus ***Wnt1*** gene is expressed in the blastula (blast) in the presumptive posterior pole, around the blastopore in gastrulae (G3-G7) and in the posterior wall of the neurenteric canal of neurulae (N2-N3) and early larvae (L1). ***Wnt8*** is expressed in a ring in blastulae, in paraxial mesendoderm in G3-G7, in paraxial mesoderm (so) in N2-N3, posterior ventral endoderm in N2-L1, neurectoderm in N3 (nt) and L1 (cv) and the presumptive mouth and anterior endoderm (endostyle and Hatschek's left diverticulum) in L1. ***Wnt11*** is expressed in the blastula, posterior mesendoderm in G3-G7, posterior somitic mesoderm and ventral posterior endoderm in N1-N3, and anterior endoderm derivatives, posterior ventral endoderm, fin ectoderm (fi) and left anteroventral ectoderm in L1. ***Wnt3*** is expressed around the blastopore in G3-G7 including dorsal blastopore lip, and neurectoderm (nt) and posterior ectoderm in N2-L1. In L1, neurectoderm expression includes the cv. ***Wnt4*** is expressed throughout mesendoderm in G3, but more strongly inside the blastopore, in posterior paraxial mesoderm and hindgut endoderm from G7-N3, as well as neurectoderm (cv and nt) in N3 and L1. In L1, expression can be clearly seen in the tailbud and anterior ventral mesothelial cells (cross section). ***Wnt5*** is expressed in mesendoderm, most strongly at the dorsal blastopore until G7, in posterior paraxial mesoderm in N3, in anterior endoderm and neurectoderm (cv) in N3 and L1, as well as the tailbud (tb) including chordoneural hinge and posterior neurenteric canal. By L1 expression can also clearly be seen in the anterior notochord (inset). ***Wnt6*** is expressed between G3 and G7 around the blastopore and in mesendoderm, in isolated spots in the neural plate and neural tube and posterior lateral mesoderm from N3 until L1. ***Wnt7*** is expressed in the neural tube from N3 to L1, including the cerebral vesicle (cv). ***Wnt16*** is expressed in paraxial mesoderm of forming somites, excluding the first pair, from N3 and in last formed somites of L1. ***Wnt10*** is expressed in posterior ectoderm and lateral paraxial mesoderm from N3, and in dorso-lateral mesoderm of somites, and in isolated anterior endodermal cells and neurectodermal spots (cv and nt) in L1. ***Wnt2*** is expressed in

axial mesoderm and neural tube in N3; a spot in the cv, anterior notochord and anterior ventral endoderm in N3, and in the anterior and posterior notochord and anterior endoderm of the rostral coelom in L1. ***Wnt9*** is expressed in ventral endoderm in N3 and endodermal derivatives including the first gill slit and endostyle primordium, left Haetschek's diverticulum, and at the site of the future mouth in L1. A weak spot of expression in the cv begins in L1. Finally, ***WntA*** is expressed in spots under the ectoderm on the left side in N3, and in the mouth primordium, cv and ventral mesothelial cells in L1.

Text S2. References for Figure S3. Specific references cited were used alongside expression database searches as per Materials and Methods (asterisks); expression data from databases are not always derived from primary literature but may have been deposited directly.

1. Abitua PB, Wagner E, Navarrete IA, Levine M (2012) Identification of a rudimentary neural crest in a non-vertebrate chordate. Nature. 2012 Dec 6;492(7427):104-7.
2. * Antin PB, Yatskievych TA, Davey S, Darnell DK (2014) GEISHA: an evolving gene expression resource for the chicken embryo. *Nucleic Acids Res.* 42(Database issue):D933-7.
3. Barriga EH, Trainor PA, Bronner M, Mayor R (2015) Animal models for studying neural crest development: is the mouse different? *Development.* 142(9):1555-60.
4. Beretta CA, Brinkmann I, Carl M (2011). All four zebrafish Wnt7 genes are expressed during early brain development. *Gene Expr Patterns*. 11(3-4):277-84.
5. Bi Y, Huang J, He Y, Zhu GH, Su Y, He BC, Luo J, Wang Y, Kang Q, Luo Q, Chen L, Zuo GW, Jiang W, Liu B, Shi Q, Tang M, Zhang BQ, Weng Y, Huang A, Zhou L, Feng T, Luu HH, Haydon RC, He TC, Tang N (2009) Wnt antagonist SFRP3 inhibits the differentiation of mouse hepatic progenitor cells. *J Cell Biochem*. 108(1):295-303.
6. Blader P, Strähle U, Ingham PW (1996). Three *Wnt* genes expressed in a wide variety of tissues during development of the zebrafish, *Danio rerio*: developmental and evolutionary perspectives. *Dev Genes Evol.* 206(1):3-13.
7. * Bowes JB, Snyder KA, Segerdell E, Jarabek CJ, Azam K, Zorn AM, Vize PD (2010) Xenbase: gene expression and improved integration. *Nucleic Acids Res.* 38:D607-D612.
8. * Brozovic M, Martin C, Dantec C, Dauga D, Mendez M, Simion P, Percher M, Laporte B, Scornavacca C, Di Gregorio A, Fujiwara S, Gineste M, Lowe EK, Piette J, Racioppi C, Ristoratore F, Sasakura Y, Takatori N, Brown TC, Delsuc F,

Douzery E, Gissi C, McDougall A, Nishida H, Sawada H, Swalla BJ, Yasuo H, Lemaire P (2016) ANISEED 2015: a digital framework for the comparative developmental biology of ascidians. *Nucleic Acids Res*. 44(D1):D808-18.

9. Buckles GR, Thorpe CJ, Ramel MC, Lekven AC (2004). Combinatorial Wnt control of zebrafish midbrain-hindbrain boundary formation. *Mech Dev.* 121(5):437-47.
10. Chawengsaksophak K, Svingen T, Ng ET, Epp T, Spiller CM, Clark C, Cooper H, Koopman P (2012). Loss of *Wnt5a* disrupts primordial germ cell migration and male sexual development in mice. *Biol Reprod*. 86:1–12.
11. Cox AA, Jezewski PA, Fang PK, Payne-Ferreira TL (2010) Zebrafish *Wnt9a*,*9b* paralog comparisons suggest ancestral roles for *Wnt9* in neural, oral-pharyngeal ectoderm and mesendoderm. *Gene Expr Patterns*. 10(6):251-8.
12. Cunningham TJ, Kumar S, Yamaguchi TP, Duester G (2015) Wnt8a and Wnt3a cooperate in the axial stem cell niche to promote mammalian body axis extension. *Dev Dyn*. 244(6):797-807.
13. Curtin E, Hickey G, Kamel G, Davidson AJ, Liao EC (2011) Zebrafish *wnt9a* is expressed in pharyngeal ectoderm and is required for palate and lower jaw development. *Mech Dev*. 128(1-2):104-15.
14. Di Maio A, Setar L, Tiozzo S, De Tomaso AW (2015) Wnt affects symmetry and morphogenesis during post-embryonic development in colonial chordates. *Evodevo*. 6:17.
15. Duncan RN, Panahi S, Piotrowski T, Dorsky RI (2015). Identification of *Wnt* genes expressed in neural progenitor zones during zebrafish brain development. *PLoS One*. 10(12):e0145810.
16. Eisenberg LM, Eisenberg CA (2006) *Wnt* signal transduction and the formation of the myocardium. *Dev Biol*. 293(2):305-15.
17. * Finger JH, Smith CM, Hayamizu TF, McCright IJ, Xu J, Law M, Shaw DR, Baldarelli RM, Beal JS, Blodgett O, Campbell JW, Corbani LE, Lewis JR,

Forthofer KL, Frost PJ, Giannatto SC, Hutchins LN, Miers DB, Motenko H, Stone KR, Eppig JT, Kadin JA, Richardson JE, Ringwald M (2017) The mouse Gene Expression Database (GXD): 2017 update. *Nucleic Acids Res.* 45 (D1): D730-D736.

18. García-Castro MI, Marcelle C, Bronner-Fraser M (2002) Ectodermal Wnt function as a neural crest inducer. *Science.* 297(5582):848-51.
19. Garriock RJ, Warkman AS, Meadows SM, D'Agostino S, Krieg PA (2007) Census of vertebrate *Wnt* genes: isolation and developmental expression of *Xenopus Wnt2, Wnt3, Wnt9a, Wnt9b, Wnt10a*, and *Wnt16. Dev Dyn.* 236(5):1249-58.
20. Geetha-Loganathan P, Nimmagadda S, Antoni L, Fu K, Whiting CJ, Francis-West P, Richman JM (2009) Expression of *WNT* signalling pathway genes during chicken craniofacial development. *Dev Dyn*. 238(5):1150-65.
21. Gessert S, Kühl M (2010) The multiple phases and faces of *wnt* signaling during cardiac differentiation and development. *Circ Res.* 107(2):186-99.
22. Hamilton PW, Sun Y, Henry JJ (2016) Lens regeneration from the cornea requires suppression of Wnt/β-catenin signaling. *Exp Eye Res*. 145:206-215.
23. Heasman J, Wessely O, Langland R, Craig EJ, Kessler DS (2001) Vegetal localization of maternal mRNAs is disrupted by VegT depletion. *Dev Biol.* 240(2):377-86.
24. Hollyday M, McMahon JA, McMahon AP (1995) *Wnt* expression patterns in chick embryo nervous system. *Mech Dev*. 52(1):9-25.
25. * Howe DG, Bradford YM, Conlin T, Eagle AE, Fashena D, Frazer K, Knight J, Mani P, Martin R, Moxon SA, Paddock H, Pich C, Ramachandran S, Ruef BJ, Ruzicka L, Schaper K, Shao X, Singer A, Sprunger B, Van Slyke CE, Westerfield M (2013) ZFIN, the Zebrafish Model Organism Database: increased support for mutants and transgenics. *Nucleic Acids Res*. 41(Database issue):D854-60.

26. Imai KS, Hino K, Yagi K, Satoh N, Satou Y (2004) Gene expression profiles of transcription factors and signaling molecules in the ascidian embryo: towards a comprehensive understanding of gene networks. *Development.* 131(16):4047-58.

27. Kataoka K, Tazaki A, Kitayama A, Ueno N, Watanabe K, Mochii M (2005) Identification of asymmetrically localized transcripts along the animal-vegetal axis of the *Xenopus* egg. *Dev Growth Differ*. 47(8):511-21.

28. Kelly GM, Lai CJ, Moon RT (1993) Expression of *wnt10a* in the central nervous system of developing zebrafish. *Dev Biol.* 158(1):113-21.

29. Kemp C, Willems E, Abdo S, Lambiv L, Leyns L (2005) Expression of all *Wnt* genes and their secreted antagonists during mouse blastocyst and postimplantation development. *Dev Dyn.* 233(3):1064-75.

30. Koscielny G, Yaikhom G, Iyer V, Meehan TF, Morgan H, Atienza-Herrero J, Blake A, Chen CK, Easty R, Di Fenza A, Fiegel T, Grifiths M, Horne A, Karp NA,

31. Kurbatova N, Mason JC, Matthews P, Oakley DJ, Qazi A, Regnart J, Retha A, Santos LA, Sneddon DJ, Warren J, Westerberg H, Wilson RJ, Melvin DG, Smedley D, Brown SD, Flicek P, Skarnes WC, Mallon AM, Parkinson H (2014) The International Mouse Phenotyping Consortium Web Portal, a unified point of access for knockout mice and related phenotyping data. *Nucleic Acids Res*. 42(Database issue):D802-9.

32. Kwon C, Arnold J, Hsiao EC, Taketo MM, Conklin BR, Srivastava D (2007) Canonical Wnt signaling is a positive regulator of mammalian cardiac progenitors. *Proc Natl Acad Sci U S A*. 104(26):10894-9.

33. Lee HC, Lim S, Han JY (2016) Wnt/β-catenin signaling pathway activation is required for proliferation of chicken primordial germ cells in vitro. *Sci Rep.* 6:34510.

34. Lekven AC, Buckles GR, Kostakis N, Moon RT (2003) *Wnt1* and *wnt10b* function redundantly at the zebrafish midbrain-hindbrain boundary. *Dev Biol.* 254(2):172-87.

35. Lewis JL, Bonner J, Modrell M, Ragland JW, Moon RT, Dorsky RI, Raible DW (2004) Reiterated Wnt signaling during zebrafish neural crest development. *Development.* 131(6):1299-308.

36. Liu P, Wakamiya M, Shea MJ, Albrecht U, Behringer RR, Bradley A (1999) Requirement for Wnt3 in vertebrate axis formation. *Nat Genet*. 22(4):361-5.

37. Lush ME, Piotrowski T (2014) ErbB expressing Schwann cells control lateral line progenitor cells via non-cell-autonomous regulation of Wnt/β-catenin. *Elife*. 3:e01832.

38. Lu FI, Thisse C, Thisse B (2011) Identification and mechanism of regulation of the zebrafish dorsal determinant. *Proc Natl Acad Sci U S A*. 108(38):15876-80.

39. Niwano T, Takatori N, Kumano G, Nishida H (2009) *Wnt5* is required for notochord cell intercalation in the ascidian *Halocynthia roretzi. Biol Cell*. 101(11):645-59.

40. Makita R, Mizuno T, Koshida S, Kuroiwa A, Takeda H (1998) Zebrafish *wnt11*: pattern and regulation of the expression by the yolk cell and No tail activity. *Mech Dev*. 71(1-2):165-76.

41. Martin A, Maher S, Summerhurst K, Davidson D, Murphy P (2012) Differential deployment of paralogous *Wnt* genes in the mouse and chick embryo during development. *Evol Dev.* 14(2):178-95.

42. Matsui T, Raya A, Kawakami Y, Callol-Massot C, Capdevila J, Rodríguez-Esteban C, Izpisúa Belmonte JC (2005) Noncanonical Wnt signaling regulates midline convergence of organ primordia during zebrafish development. *Genes Dev*. 19(1):164-75.

43. Miya T, Nishida H (2002). Isolation of cDNA clones for mRNAs transcribed zygotically during cleavage in the ascidian, *Halocynthia roretzi*. *Dev Genes Evol*. 212(1):30-7.

44. Mwafi N, Beretta CA, Paolini A, Carl M (2014) Divergent *Wnt8a* gene expression in teleosts. *PLoS One*. 9(1):e85303.

45. Narita T, Sasaoka S, Udagawa K, Ohyama T, Wada N, Nishimatsu S, Takada S, Nohno T (2005) *Wnt10a* is involved in AER formation during chick limb development. *Dev Dyn*. 233(2):282-7.

46. Ohyama T, Mohamed OA, Taketo MM, Dufort D, Groves AK (2006). Wnt signals mediate a fate decision between otic placode and epidermis. *Development.* 133(5):865-75.

47. Patthey C, Clifford H, Haerty W, Ponting CP, Shimeld SM, Begbie J (2016). Identification of molecular signatures specific for distinct cranial sensory ganglia in the developing chick. *Neural Dev*. 11:3.

48. Person AD, Garriock RJ, Krieg PA, Runyan RB, Klewer SE (2005) Frzb modulates Wnt-9a-mediated beta-catenin signaling during avian atrioventricular cardiac cushion development. *Dev Biol.* 278(1):35-48.

49. Poulain M, Ober EA (2011) Interplay between *Wnt2* and *Wnt2bb* controls multiple steps of early foregut-derived organ development. *Development*. 138(16):3557-68.

50. Pradhan A, Olsson PE (2014) Juvenile ovary to testis transition in zebrafish involves inhibition of *ptges*. *Biol Reprod*. 91(2):33.

51. Quinlan R, Graf M, Mason I, Lumsden A, Kiecker C (2009) Complex and dynamic patterns of *Wnt* pathway gene expression in the developing chick forebrain. *Neural Dev*. 4:35.

52. * Richardson L, Venkataraman S, Stevenson P, Yang Y, Moss J, Graham L, Burton N, Hill B, Rao J, Baldock RA, Armit C. (2014) EMAGE mouse embryo

spatial gene expression database: (2014 update). *Nucleic Acids Res.* 42(1):D835-44.

53. Saitou M, Yamaji M (2012) Primordial germ cells in mice. *Cold Spring Harb Perspect Biol.* 4: a008375.
54. Sasakura Y, Ogasawara M, Makabe KW (1998) *HrWnt-5*: a maternally expressed ascidian *Wnt* gene with posterior localization in early embryos. *Int J Dev Biol*. 42(4):573-9.
55. Sasakura Y, Makabe KW (2000) Ascidian *Wnt-7* gene is expressed exclusively in the tail neural tube of tailbud embryos. *Dev Genes Evol*. 210(12):641-3.
56. Sienknecht UJ, Fekete DM (2008) Comprehensive Wnt-related gene expression during cochlear duct development in chicken. *J Comp Neurol*. 510(4):378-95.
57. Sienknecht UJ, Fekete DM (2009) Mapping of *Wnt, frizzled*, and *Wnt* inhibitor gene expression domains in the avian otic primordium. *J Comp Neurol*. 517(6):751-64.
58. Steventon B, Mayor R, Streit A (2014) Neural crest and placode interaction during the development of the cranial sensory system. *Dev Biol.* 389(1):28-38.
59. Summerhurst K, Stark M, Sharpe J, Davidson D, Murphy P (2008) 3D representation of *Wnt* and *Frizzled* gene expression patterns in the mouse embryo at embryonic day 11.5 (Ts19). *Gene Expr Patterns*. 8(5):331-48.
60. Takada S, Stark KL, Shea MJ, Vassileva G, McMahon JA, McMahon AP (1994) *Wnt-3a* regulates somite and tailbud formation in the mouse embryo. *Genes Dev.* 8(2):174-89.
61. Tanaka SS, Nakane A, Yamaguchi YL, Terabayashi T, Abe T, Nakao K, Asashima M, Steiner KA, Tam PP, Nishinakamura R. Dullard/Ctdnep1 modulates WNT signalling activity for the formation of primordial germ cells in the mouse embryo (2013) *PLoS One*. 8(3):e57428.
62. Tevosian SG (2012) Gone without the WNT: a requirement for WNT5A in germ cell migration and testis development. *Biol Reprod*. 86(1):1-2.

63. Tao Q, Yokota C, Puck H, Kofron M, Birsoy B, Yan D, Asashima M, Wylie CC, Lin X, Heasman J (2005) Maternal *wnt11* activates the canonical wnt signaling pathway required for axis formation in *Xenopus* embryos. *Cell.* 120(6):857-71.
64. Ungar AR(1), Kelly GM, Moon RT (1995) *Wnt4* affects morphogenesis when misexpressed in the zebrafish embryo. *Mech Dev*. 52(2-3):153-64.
65. Vainio S, Heikkilä M, Kispert A, Chin N, McMahon AP (1999) Female development in mammals is regulated by Wnt-4 signalling. *Nature*. 397(6718):405-9.
66. Visel A, Thaller C, Eichele G (2004) GenePaint.org: an atlas of gene expression patterns in the mouse embryo. *Nucleic Acids Res*. 32 (Database issue):D552-6.
67. Witte F, Dokas J, Neuendorf F, Mundlos S, Stricker S (2009) Comprehensive expression analysis of all *Wnt* genes and their major secreted antagonists during mouse limb development and cartilage differentiation. *Gene Expr Patterns* 9(4):215-23.
68. Yagi K, Satoh N, Satou Y. Identification of downstream genes of the ascidian
69. muscle determinant gene *Ci-macho1* (2004) *Dev Biol*. 274(2):478-89.
70. Zhang B, Tran U, Wessely O (2011) Expression of *Wnt* signaling components during *Xenopus* pronephros development. *PLoS One*. 6(10):e26533.
